# Barriers and enablers to diabetic eye screening attendance: An interview study with young adults with type 1 diabetes

**DOI:** 10.1111/dme.14751

**Published:** 2021-12-29

**Authors:** Louise Prothero, John G. Lawrenson, Martin Cartwright, Roxanne Crosby‐Nwaobi, Jennifer M. Burr, Philip Gardner, John Anderson, Justin Presseau, Noah Ivers, Jeremy M. Grimshaw, Fabiana Lorencatto, for the EROS Study Investigators

**Affiliations:** ^1^ School of Health Sciences, City University of London London UK; ^2^ NIHR Moorfields Biomedical Research Centre London UK; ^3^ School of Medicine University of St Andrews St Andrews UK; ^4^ Office for Health Improvement and Disparities Department of Health and Social Care London UK; ^5^ Homerton University Hospital NHS Foundation Trust London UK; ^6^ Ottawa Hospital Research Institute Ottawa Ontario Canada; ^7^ Women’s College Research Institute Toronto Ontario Canada; ^8^ Department of Medicine University of Ottawa Ottawa Ontario Canada; ^9^ Centre for Behaviour Change University College London London UK

**Keywords:** barriers and enablers, behaviour change, diabetic eye screening, qualitative research

## Abstract

**Aim:**

The aim of this study was to identify barriers and enablers of diabetic eye screening (DES) attendance amongst young adults with diabetes living in the United Kingdom.

**Methods:**

Semistructured qualitative interviews with adults aged 18–34 years with diabetes. Participants were purposively sampled to aim for representation across gender, geographical locations, diabetes type, years since diabetes diagnosis and patterns of attendance (i.e. regular attenders, occasional non‐attenders, regular non‐attenders). Data were collected and analysed using the Theoretical Domains Framework (TDF) to explore potential individual, sociocultural and environmental influences on attendance. Data were analysed using a combined deductive and inductive thematic analysis approach. Barriers/enablers were mapped to behaviour change techniques (BCTs) to identify potential strategies to increase attendance.

**Results:**

Key barriers to attendance reported by the sample of 29 study participants with type 1 diabetes, fell within the TDF domains: [*Knowledge*] (e.g. not understanding reasons for attending DES or treatments available if diabetic retinopathy is detected), [*Social Influences*] (e.g. lack of support following DES results), [*Social role and Identity*] (e.g. not knowing other people their age with diabetes, feeling ‘isolated’ and being reluctant to disclose their diabetes) and [*Environmental Context and Resources*] (e.g. lack of appointment flexibility and options for rescheduling). Enablers included: [*Social Influences*] (e.g. support of family/diabetes team), [*Goals*] (e.g. DES regarded as ‘high priority’). Many of the reported barriers/enablers were consistent across groups. Potential BCTs to support attendance include *Instructions on how to perform the behaviour*; *Information about health consequences*; *Social support (practical)* and *Social comparison*.

**Conclusions:**

Attendance to diabetic eye screening in young adults is influenced by a complex set of interacting factors. Identification of potentially modifiable target behaviours provides a basis for designing more effective, tailored interventions to help young adults regularly attend eye screening and prevent avoidable vision loss.

**What’s new?**

- An organised screening programme for diabetic eye disease reduces the risk of visual impairment, but attendance is suboptimal, particularly in adults aged 18–34 years.
- Previous studies have explored modifiable influences on screening attendance, but often do not differentiate between population groups, few studies focus on young adults.
- We applied the Theoretical Domains Framework to identify modifiable barriers and enablers to screening attendance in young adults with type 1 diabetes living in the United Kingdom.
- Common barriers included lack of understanding of diabetic eye disease or its treatment; lack of appointment flexibility and the need for information and support following screening results. Social support of family and the diabetes team were identified as a key enabler.
- These findings provide a basis for developing more targeted interventions. Potential strategies to increase attendance in this group include tailored education, persuasive communication and integration of diabetic eye screening with other diabetes appointments

## 1 BACKGROUND

In the United Kingdom, diabetic eye screening (DES) is managed by the National Screening Committee.^1^ In England, the NHS National Diabetic Eye Screening Programme provides annual screening for approximately 3.3 million eligible people with diabetes aged 12 years and over through 57 regional Diabetic Eye Screening Programmes (DESPs). Equivalent National programmes in Scotland, Wales and Northern Ireland operate according to similar service specifications. Although uptake of screening is generally high (82.6% in England 2018–2019), published audits report significant inequity in screening attendance and outcomes, with variable uptake and suboptimal attendance in particular demographic groups.^2^, ^3^, ^4^, ^5^ One such group is young adults with diabetes. A recent retrospective analysis of attendance in three large screening programmes in England identified that the odds of attending annual screening were significantly lower amongst those aged 18–34 years compared to those ≥60, after controlling for other variables, for example, sex, ethnicity and socioeconomic deprivation.^4^

This raises questions as to how screening attendance can be increased in young adults to prevent complications and avoidable vision loss. To answer this, we must first understand the reasons why young adults attend or do not attend DES (i.e. the barriers and enablers). There have been a number of studies internationally exploring barriers/enablers to diabetic retinopathy screening, which are summarised in a recent systematic review.^6^ However, the majority of the included studies treated people with diabetes as a homogeneous group and typically did not explore barriers and enablers to attendance from the perspective of particular demographic groups and/or those who have evidenced lower attendance relative to all people with diabetes.^6^ For example, the review identified only two qualitative studies exploring barriers/enablers to DES in young adults.^7^, ^8^ Although further studies have since been published on barriers to screening attendance in this population,^9^, ^10^ the evidence base remains sparse, particularly the availability of theory‐based studies of barriers and enablers to DES attendance in young adults.

Delivery of DES by healthcare professionals (HCPs) and attendance at screening appointments are examples of complex human behaviour. Theories provide explicit statements regarding processes hypothesised to regulate behaviour, and can be used to explain and predict human behaviours.^11^ The use of a theory enables drawing from, and contributing to, the decades of evidence in the wider literature regarding what influences behaviour and how best to change it. However, there are many, overlapping behaviour change theories. One behavioural science framework, the Theoretical Domains Framework (TDF), synthesises constructs from 33 behaviour change theories into 14 domains representing individual, sociocultural and environmental influences on behaviours.^12^ Using the TDF to guide data collection and analysis can help ensure the broad range of potential barriers and enablers to the behaviour of interested is identified. A strength of the TDF is that it is linked to two complementary frameworks for specifying different types of interventions and techniques that can be used to change behaviour (i.e. the Behaviour Change Wheel^13^ and the Behaviour Change Technique (BCT) Taxonomy).^14^ This facilitates the systematic progression from understanding what is driving behaviour to designing more targeted strategies to change behaviour and therefore linking barriers to solutions. In the context of DES, the TDF has been recently used to explore barriers/enablers to DES in Australian young adults with type 2 diabetes^7^ and Canadian adults from ethnic minority groups with diabetes.^15^

The aim of the current study was to apply the aforementioned behavioural science frameworks to:

1. Identify barriers and enablers to DES in young adults aged 18–34 years living in the United Kingdom.
2. Identify potential behaviour change intervention strategies to encourage attendance in this population group.

## 2 METHODS

### 2.1 Design

A behaviour change theory‐informed qualitative study of young adults with diabetes.

### 2.2 Ethical approvals

This study received ethical approval from the NHS Wales Research Ethics Committee 2 (REC reference: 19/WA/0228).

### 2.3 Participants, recruitment and sampling

Eligible participants were English speaking, adults aged 18–34 years with type 1 or type 2 diabetes, who had attended at least one DES appointment. The choice of the age range 18–34 years was based on previously published audits of the UK diabetic eye screening programme, showing that this group is least likely to attend first and subsequent retinopathy screening and was most likely to be repeat non‐attenders.^2^, ^4^, ^5^ We recruited participants by circulating a study invitation poster via social media platforms (e.g. Diabetes UK and JDRF Twitter accounts) and by sending invitation letters by mail to young adults (<35 years) on the register of a large urban screening programme. Participants were offered a £15 shopping voucher as an incentive to take part.

Purposive sampling was conducted with the aim of achieving variation within the target age group in terms of geographical location, ethnic group, type of diabetes and past history of attendance. For attendance, we retrospectively categorised participants as regular attenders (i.e. participants who have previously attended all DES appointments); unintentional non‐attenders (i.e. participants who have unintentionally forgotten/missed previous DES appointments, and have rescheduled); and intentional non‐attenders (i.e. participants who have actively chosen to not attend previous DES appointments).

The target sample size for the current study was up to 30 interviews. Recruitment continued, with concurrent analysis, until thematic saturation was reached, that is, no new themes were emerging from the data and existing themes were supported by data from several participants.^16^

### 2.4 Study materials

The semistructured interview topic guides aimed to understand reasons why young adults do or do not attend DES attendance. The topic guide was developed by a team of behavioural scientists, health psychologists and clinicians, with input from four young adults with diabetes. The questions in the topic guide were structured around the 14 domains of the TDF: *knowledge*; *skills*; *social*/*professional role and identity*; *beliefs about capabilities*; *beliefs about consequences*; *optimism*; *reinforcement*; *intention*; *goals*; *memory*/*attention*/*decision processes*; *environmental context*/*resources*; *behavioural regulation*; *social influences*; and *emotion*. The interviews were piloted prior to data collection with two young adults with diabetes, and refined accordingly to enhance clarity and flow. The final version of the topic guide is available in Appendix S1.

### 2.5 Procedure

One‐to‐one interviews were conducted via telephone by the same researcher (LP), a health psychologist with extensive experience in qualitative research. Interviews took place between December 2019 and September 2020. Participants were asked to either complete an informed consent form and send this via email to the researcher ahead of the interview, or provide verbal consent prior to the start of the interview. Interviews were audio‐recorded, transcribed verbatim and fully anonymised, so that no individual could be identified from the data.

### 2.6 Analysis

Transcripts were analysed using deductive framework analysis to code text into the broad TDF domains, followed by inductive thematic analysis to further code text within each domain.^9^ Analysis of the interview transcripts followed a stepwise process:

1. Developing a coding framework structured around the 14 TDF domains following collaborative coding of three interview transcripts by three researchers (LP/FL/MC). The coding framework is available in Appendix S2 (Table S1).
2. Generating a template summary of each interview transcript following methods for rapid qualitative analysis.^17^ A health psychology researcher (LP) independently generated a template summary for the first 14 transcripts. This involved using the codebook to deductively code data to the domain they were judged to best represent.
3. Inductive themes, sometimes referred to as ‘Belief Statements’ in the TDF literature,^11^ were developed based on the summarised data within each domain, across participants. Themes were classified as either a barrier to DES, enabler or mixed theme (influences that operate as both a barrier and an enabler for the same participant and/or across participants). Two experienced behavioural and health psychology researchers (FL and MC) independently reviewed inductively generated themes at regular intervals throughout the analysis to check whether they agreed the theme label represented the data summaries contributing to that theme, and whether it was allocated to the most appropriate TDF domain. Any discrepancies were resolved through discussion until consensus was reached.
4. A matrix was used to look for similarities, differences and trends in responses: Following analysis of all 29 interview transcripts, themes were transferred into an Excel table of respondents by domains.
5. The key domains and themes representing barriers/enablers to DES were identified using established criteria: (a) frequency (the number of participants whose responses contributed to that theme, particularly the number of non‐attending participants); (b) spontaneity (did the theme occur spontaneously or was it elicited by a question in the topic guide); (c) elaboration (number of themes per domain).^6^, ^11^

### 2.7 Mapping to intervention strategies

Using a similar approach to van Allen et al.,^15^ themes generated through the qualitative analysis representing barriers/enablers to DES were mapped to potential intervention approaches using available mapping tools,^18^ previous evidence from the literature and stakeholder consultation. Mapping tools suggest which intervention strategies are more likely to be appropriate for addressing barriers and enablers within different domains of the TDF^12^; thereby providing a basis for systematically progressing from initial identification of ‘what needs to change' to selecting potential intervention components for further iterative development of intervention content.^13^ For each TDF domain, the mapping tools were consulted to identify potential techniques that have been established as being appropriate for addressing the barriers/enablers identified within that domain during the qualitative analysis. To select amongst the candidate list of potential techniques, we consulted a Cochrane Review of interventions to increase DES.^19^ Suggested interventions were then summarised in an intervention mapping table, which was then shared with a stakeholder advisory group consisting of diabetologists, ophthalmologists, screener/graders, young adults with diabetes, policy and diabetes charity representatives, who were invited to comment on the proposed interventions, provide suggestions for refinements in how the intervention might be delivered or additional suggestions for intervention strategies.

## 3 RESULTS

### 3.1 Participants

We conducted interviews with young adults with type 1 diabetes, lasting an average of 30 min (range 12–50 min). We were unable to recruit participants within our 18–34 age group with type 2 diabetes.

Data saturation was deemed achieved after 29 interviews. Fifteen of these participants were regular DES attenders, six were unintentional non‐attenders and eight were intentional non‐attenders. Other participant demographic characteristics are summarised in Table 1.

**TABLE 1:** Participant demographics

	% (n)
Gender	
Women	62.1 (18)
Men	37.9 (11)
Age (years)	
18–23	31.0 (9)
24–29	38.0 (11)
30–35	31.0 (9)
Duration of diabetes (years)	
18–26	41.4 (12)
9–17	13.8 (4)
1–8	44.8 (13)
Ethnicity	
White British	79.3 (23)
White European	6.9% (2)
White and Black Caribbean	3.4% (1)
Irish	3.4% (1)
Caribbean	3.4% (1)
Any other ethnic group	3.4% (1)
Country of residence	
England	75.9 (22)
Northern Ireland	13.8 (4)
Scotland	6.9 (2)
Wales	3.4 (1)
Area	
Urban	37.9 (11)
Suburban	34.5 (10)
Rural	27.6 (8)
Occupational status	
Full‐time job	58.6 (17)
Part‐time job	6.9 (2)
Studying full‐time	17.2 (5)
Studying part‐time	6.9 (2)
Unemployed	6.9 (2)
Other—Freelancer	3.4% (1)
Highest level of education	
School education (up to 16)	6.9 (2)
Further education (up to 18)	34.5 (10)
Bachelor's degree or more	58.6 (17)

### 3.2 Barriers and enablers to DES

Reported barriers and enablers to DES attendance were identified across 13 (of 14) domains, with the exception of *Optimism*. Table 2 ranks domains in terms of relevance to the behaviour in terms frequency, elaboration and spontaneity of themes.

**TABLE 2:** Domain importance

Domain	Frequency (max n=29) (number of participants reporting barriers or enablers within the domain)	Elaboration (number of barrier or enabler themes per domain)	Spontaneity (Frequency of spontaneous themes)
Knowledge	29	7	4
Social/professional role and identity	29	5	3
Environmental Context & Resources	28	10	6
Social influences	28	8	8
Goals	27	3	2
Intention	27	2	1
Memory, attention and decision processes	26	7	2
Emotion	26	5	5
Beliefs about consequences	24	5	4
Skills	24	2	0
Reinforcement	20	2	0
Beliefs about capabilities	13	1	1
Behavioural Regulation	3	1	1

Table 3 provides an example of a subset of themes representing barriers and enablers that were identified within each domain. A narrative description of the themes, within domains, is presented below for the domains that were identified as highly relevant (based on elaboration and spontaneity), highlighting any key differences according to attendance status. The full list of themes (i.e. barriers/enablers) identified within each domain is presented in Appendix S3 (Table S2), along with supporting quotes.

**TABLE 3:** Barriers and enablers to DES identified within each TDF domain

Theme	Barrier/Enabler/Mixed	Frequency (total max N=29)			Example quote(s)
		Regular attenders (N=15)	Un‐intentional non‐attenders (N=6)	Non‐attenders (N=8)	
TDF domain: KNOWLEDGE					
Not knowing a lot about the treatments available if DR is detected	Barrier	12	5	5	*‘I don't really know much, I just know that either they keep an eye on it for a few years, or you keep going in to see a specialist instead of the regular screenings and then if it gets worse I think it starts with the laser and then a couple of operations can be done as well I think, but I don't really know that much about what's done’. (Barrier)*
(Not) understanding the reasons for attending DES	Mixed	13	5	8	*‘I think it's just they can just check to see if there's any changes in your eyes so from the pictures and if they notice anything different then they can follow it up as soon as it happens instead of waiting until it gets worse’. (Enabler)* *‘I didn't feel that I was given any education on why my screenings were important in the first year or two. So I went to my first one, didn't go to my second’. (Barrier)*
Awareness of diabetes and DES	Mixed	7	0	3	*‘I do think there is a bit of a lack of awareness, even with the people doing the screenings, might not know that younger people can be diabetic as well’. (Barrier)* *‘I think maybe just a bit more awareness of it, you know if a leaflet came out or something with the letter or even if there was more on social media about it. It might just make me more aware of the effects of if I don't go to these screenings’. (Barrier)*
TDF domain: SKILLS					
Diabetes education/training did not cover DES in detail	Barrier	11	2	7	*‘It probably did. It would've been brief though. In, yeah, a couple of things that I’ve been to talks about it maybe for ten minutes’. (Barrier)*
TDF domain: SOCIAL/PROFESSIONAL ROLE AND IDENTITY					
Feeling isolated or different during teenage years	Barrier	3	1	3	*‘[…] just going through the teenage years was really difficult, just trying to fit in at school and manage Type 1 […] I would say it's pretty isolating because you feel like you're going through everything on your own’. (Barrier)*
Willingness to disclose diabetes	Mixed	14	6	8	*‘Very reluctant, I’m renowned for being somebody who keeps it quite secret, until I know somebody well’. (Barrier)* *‘I don't mind at all because I like to educate them a bit if they don't know anything’. (Enabler)* *‘At first, when I was six years old, I felt a bit weird talking about it but, as I’ve got older, I’ve just accepted it’. (Barrier then Enabler)*
Do (not) know other people their age with diabetes	Mixed	15	4	8	*‘No. I don't know anyone’. (Barrier)* *‘So, in my area when I was younger, they used to send us off on diabetic camp’. (Enabler)*
TDF domain: BELIEFS ABOUT CAPABILITIES					
Having well‐controlled diabetes/blood sugars	Enabler	9	3	1	*‘I am really well controlled, so I don't really need to see them [DSN, diabetes specialist nurse] or speak to them that much’. (Enabler)*
TDF domain: BELIEFS ABOUT CONSEQUENCES					
Negative impact of eye drops	Barrier	4	2	3	*‘I just remember obviously coming out afterwards and I was walking down the corridor and I just couldn't see a thing and I couldn't see my phone to ring my grandma or anything. So I was, I know so I was walking down I must have looked like an absolute crazy person’. (Barrier)*
Attend DES to avoid DR, to monitor eyes and for the early detection of complications	Enabler	8	5	4	*‘So, I am trying to obviously go to my [3] appointments and stuff like that, so that I can avoid getting it [DR] […] Because I don't want to be end up not being able to see’. (Enabler)*
Importance of eyesight	Enabler	2	2	1	*‘[DR a concern] Because I do believe its, my eyes are really, really valuable to me. I read a lot’. (Enabler)*
TDF domain: REINFORCEMENT					
Mixed feelings of pressure to attend DES	Mixed	10	4	6	*‘No, no pressure, no.’ (Barrier)* *‘Only pressure that I put on myself’*. *(Enabler*)
TDF domain: INTENTION					
Strong intention to attend future DES appointments	Enabler	15	5	7	*‘Very likely. I don't intend to miss any of them’. (Enabler)*
TDF domain: GOALS					
Priorities in diabetes management	Enabler	12	6	7	*‘I think for my diabetes management, I think the main thing for me is literally just managing my glucose levels and, day to day […] it's my diet and stuff like that. Staying fit and making sure that I don't get any complications further down the line with retinopathy or heart disease or anything associated with that’. (Enabler)*
Attending DES becomes more of a priority when experienced complications	Enabler	2	2	2	*‘If I hadn't had that past experience, if someone said to me, [participant name], it's going to take five hours out of your day or, I’d probably go, (sighs) well I’ll go next week and then I’d probably forget about it’. (Enabler)*
TDF domain: MEMORY, ATTENTION AND DECISION‐MAKING					
Forgetting to attend at least one DES appointment	Barrier	5	4	1	*‘I’ve never actively chosen not to [attend DES]. I think I just might have forgotten to go to it but I’ve never […] it's not a case of, I’m not getting any value from this’. (Barrier)*
Prompts and reminders to attend	Enabler	5	3	2	*‘Probably a text [would have helped remind her to attend], because people do check their phones more often now I think. So I think a text reminder would have helped’. (Barrier)*
Preference to receive appointment information by text/e‐mail/phone call, instead of by letter	Enabler	3	2	3	*‘I travel quite a lot with work so there might be times where I miss the letter, or my parents, I live with my parents and they're terrible for letting letters pile up and it will get put to the bottom of that pile [….] so if they were to send a letter, an email or a text message, or even just give me call that would be really useful’. (Enabler)*
TDF domain: ENVIRONMENTAL CONTEXT/RESOURCES					
DES appointments take up half a day or more	Barrier	6	3	5	*‘[…] if you include the drops, probably three to four hours until your eyesight's fully back to normal’. (Barrier)*
Need for more flexibility and options for (re‐) scheduling DES appointments	Barrier	7	3	4	*‘Just another option of day, just a, yeah, just a second option just to say, look this is the other day that we can do. Just so that I can make the best decision for me around my schedule’. (Barrier)*
DES and diabetes care are (not) co‐ordinated	Mixed	7	5	5	*‘They're just, all my appointments are just random. Nothing lines up it's all just, they just come at random’. (Barrier)* *‘Usually pretty well actually. They dovetail quite nicely in between my consultant appointments’. (Enabler)*
TDF domain: SOCIAL INFLUENCES					
Seeing older people with worse complications in the DES waiting room	Barrier	2	1	0	*‘I think awareness in the sort of environment you going to […] I find that every time I go it's just full of really old people that have not necessarily taken the best care of their diabetes. So a lot of the time I’ll go and sit there and there'll be people without their legs and stuff and I just find it not very pleasant to sit in the waiting room and look around’. (Barrier)*
Need for more support and information following DES results	Barrier	6	2	4	*‘[…] you don't need someone saying like you're doing a shit job. What you need is […] you need someone to explain to you, OK, this is what we found, or this is what potentially could happen. These are the options, don't worry about it, do you know what I mean?’ (Barrier)*
Impact of healthcare professional communication	Mixed	11	1	2	*‘When I got told I had the macular oedema thing my GP was like, you're a bit young to have any diabetic complications, and it's like, well I have been diabetic 20 years, that's the same as someone getting it when they're 40 and seeing complications when they're 60, it's just, yeah. So, I think there's a bit of a gap where even medical professionals don't really think about what they're saying sometimes’. (Barrier)* *‘I think for me, there's something about knowing that I’ve got a team behind me, that's there to help me that makes me think, I want to attend that, I want to be there, I want to be part of it. I think that's a huge focus for me, so just that communication between a diabetic and whoever's the healthcare professionals, I think it's just really important’. (Barrier)*
TDF domain: EMOTION					
Diabetes distress/burnout	Barrier	1	0	3	*‘So I’ve literally been probably two years in the past just really burnt out with and overwhelmed by diabetes care and slipped into a pattern of just not taking my medication, not checking my blood glucose levels and not going to eye screening so, yeah’. (Barrier)*
DR is a concern	Enabler	9	4	6	*‘Yeah [DR a concern] I think everyone worries that they're going to lose, well, not everyone but if there's a possibility you're going to lose your sight, it's going to be a bit of a worry’. (Enabler)*
Feelings about receiving DES results	Mixed	9	4	5	*‘It depends on if they're good or bad I’d say. Yeah, usually not great about it to be honest’. (Barrier)* *‘Yeah, I feel fine. It's good to have peace of mind, I guess’. (Enabler)*
TDF domain: BEHAVIOURAL REGULATION					
Putting DES appointments in electronic calendar once they receive the appointment letter	Enabler	1	1	1	*‘I just put it in my calendar now […] a little thing in my Google calendar, yeah’. (Enabler)*

#### 3.2.1 Knowledge

*Knowledge* was a mixed influence on DES attendance. Although enablers reported in this domain included understanding both how diabetes affects the eyes and the reasons for attending DES, all but one of the non‐attenders reported that educational courses, for example, DAFNE did not cover diabetic eye disease and DES in detail. A common knowledge barrier was being unaware of the treatments available if diabetic retinopathy were to be detected. Several participants indicated that they would like to know more about the treatments available. In contrast, a small number indicated that they would not want to know more, unless treatment was required.

#### 3.2.2 Social role and identity

*Social Role and Identity* was a mixed influence on DES attendance. Barriers within this domain were particularly reported by intentional non‐attenders and included participants not knowing other people their age with diabetes, feeling ‘isolated’ and the ‘odd one out’ during school and teenage years and being reluctant to disclose their diabetes. Most intentional non‐attenders described becoming more comfortable disclosing their diabetes as they became older. In support to this, an enabler within this domain included *‘knowing others with diabetes*/*being part of the online community means you are more engaged with your diabetes management’*. A number of intentional non‐attenders described actively making steps to meet other young adults with diabetes. This resulted in them becoming more engaged with their DES appointments around their early to mid‐20s.

#### 3.2.3 Environmental context and resources

*Environmental context* and *resources* was a mixed influence on DES attendance. Common barriers within this domain included lack of flexibility and options for (re‐) scheduling DES appointments (e.g. evening/weekend appointments, appointments offered on more than 1 day) and appointments taking up to half a day or more. A further barrier was transitioning from paediatric to adult care and the impact of university. Transitioning from paediatric to adult diabetes care meant being seen less frequently in diabetes clinics and usually by an unfamiliar team and some participants ‘dropped off’ from attending DES during this ‘difficult period’. Participants experienced difficulties attending DES whilst at university; either due to delays in changing eye screening programme or having to attend DES appointments outside of term time. The lack of co‐ordination between DES and other diabetes appointments was particularly reported as a barrier by unintentional non‐attenders and included issues such as ‘random’ timing of appointments during the year and ineffective communication between the DES and diabetes care teams. The instillation of eye drops to dilate the pupil (mydriasis) is standard practice in the NHS screening programme to improve retinal image quality, although this is not always required in young adults. Advantages of not having mydriatic eye drops was an enabler to DES attendance within this domain. Specific advantages included being able to drive to and from the appointment, and DES taking less time from participants’ working day.

#### 3.2.4 Social influences

A common *Social influences* barrier was the need for more support and information after receiving DES results. Participants thought the DES results letter provided insufficient ‘blanket’ information. They reported a preference to speak to a diabetes consultant or nurse (either in‐person or over the telephone) to obtain tailored feedback (e.g. what was found, their level of risk of developing diabetic retinopathy). Impact of HCP communication was a mixed theme within this domain. Impact of HCP communication as an enabler particularly referenced diabetes specialist nurses. Participants reported nurses being ‘really helpful’ with regular contact in‐between appointments (facilitated mainly via text message and email). Communication became a barrier to DES attendance when young adults perceived HCPs displayed a lack of knowledge about diabetes, or they were being ‘judged’ or ‘spoken to harshly’ (e.g. by general practitioners or those conducting DES). Enablers to DES attendance within the *Social influences* domain include members of the diabetes team checking DES appointment attendance and partners/family members assisting with travel to and from their DES appointments. Although an enabler, participants acknowledged that requiring assistance getting to and from DES appointments was sometimes impractical (e.g. co‐ordinating a time when both they and their family member/partner were available).

#### 3.2.5 Goals

*Goal*s was an enabler to DES attendance. Priorities in diabetes management was a common theme across participants. Attending DES appointments was regarded as a high priority by the majority of participants. Attending DES became more of a priority when participants experienced eye complications. This was described by one interviewee as a ‘jolt’ that said *‘you need to sort yourself out before things get any worse’*. This theme also applied to participants with indirect experience of eye complications caused by diabetes (e.g. family members). These two interviewees separately reported feeling genetically more at risk of complications and not wanting ‘to go that way’.

#### 3.2.6 Intention

*Intention* was an enabler to DES attendance across participants. Almost all participants expressed a strong intention to attend future DES appointments. Pregnancy was a life event which increased one interviewee's likelihood of attending DES, because she knew she was more at risk of complications while pregnant.

#### 3.2.7 Memory attention and decision processes

*Memory*, *attention and decision processes* was a mixed influence on DES attendance. A common barrier within this domain was forgetting to attend at least one DES appointment. This barrier was especially reported by unintentional non‐attenders, citing issues such as DES appointments being sent to far in advance and not receiving the DES invitation letter. Enablers within this domain included preference to receive their DES appointment via text/e‐mail/phone call, rather than via a letter which can be easily missed and is also less economical. Participants suggested a text message or phone call prior to the appointment would serve as a useful reminder to attend.

#### 3.2.8 Emotion

*Emotion* was a mixed influence of DES attendance. Common enablers reported in *Emotion* included concern and worry about future diabetic eye complications. Diabetes scare stories were reported as a barrier to DES attendance. This involved young adults either reading or being warned by HCPs, about complications they will experience unless they manage their blood sugars (e.g. *‘if you don't look after yourself, you're going to go blind’*). Another barrier, reported by some intentional non‐attenders, was diabetes distress/burnout. This was caused by the burden of attending multiple appointments, the ‘constant’ demands of blood glucose management and a feeling of being ‘overwhelmed’. Mixed feelings about receiving DES results describe how interviewees felt about finding out the outcome of the screening procedure. Feeling nervous and anxious about receiving the results was especially reported by intentional non‐attenders, some of whom acknowledged that how they feel depends on the result.

### 3.3 Mapping identified barriers/enablers to intervention strategies

Table 4 presents the process of mapping barriers and enablers to proposed strategies to increase DES attendance in UK young adults for a subset of barriers and enablers identified in the interviews based on spontaneity and elaboration. The full list of suggested intervention strategies is available in Appendix S4 (Table S3). A range of potential strategies were identified. Some strategies target individual knowledge, motivational and emotional influences on DES attendance (e.g. persuasive communication, use of positive case studies and testimonials, providing reassurance around what can be done if retinopathy is detected and the benefits of screening). Some strategies operate at the service provision level (e.g. integration of DES clinics with other diabetes appointments, increasing flexibility and availability of appointments on weekends and evenings, creating opportunities for people with diabetes to discuss their test results with a HCP), while others necessitate change at the sociocultural level (e.g. improving doctor–patient communication, reducing stigma and increasing awareness about diabetes and diabetic retinopathy in the general population).

**TABLE 4:** Mapping of barriers/enablers to proposed intervention strategies.

Identified barrier/enabler	Corresponding TDF domain	Intervention function (Behaviour Change Wheel)	Behaviour Change Technique (BCT)	Proposed operationalisation of selected intervention components	Intervention priority group
**(Not) understanding the reason for attending DES (M)**— some participants previously did not understand the reasons for attending DES **Not knowing a lot about the treatments available if DR is detected (B)** **DR is a concern (E)**—main reason cited—fear of sight loss in the future	Knowledge Emotion Beliefs About Consequences	Education Persuasion	Information about health consequences^a^ Salience of consequences BCTs we do not want to deliver: anticipated regret. Need to put emphasis on the positives to minimise negative emotions rather than prompting feelings of anxiety and regret Framing/re‐framing Credible source^a^	Providing information on: 1) risks of developing DR and risks of progression using contemporary data, 2) potential complications if DR goes undetected. Emphasis placed on positive rather than negative information to minimise defensive or avoidant responses—for example, emphasise the benefits of early detection (pick things up early +can be treated). Providing information on available treatments for DR—emphasise again the positives— for example, effectiveness of treatments in helping to stop DR from progressing (particularly if caught early) Use of print media (e.g. leaflets, other written materials) with individual people and social media for use at the population level. Case studies/testimonials (e.g. video, digital resources) by other YAs with diabetes that demonstrate positive emotions and outcomes as a result of screening (i.e. I attended my screening regularly, and this meant as soon as any small changes were picked up, they could be treated straight away to stop them progressing into something more serious or sight threatening)—instead of testimonials that focus on the extremes—that is, I lost my vision/went blind because I left it too late Emphasis by screeners that attending DES reduces the risk of vision loss and YAs do not need to live in fear of future blindness Communication on reasons for attending and available treatments with HCPs (e.g. GP, Optometrist, Diabetologist)	YAs with diabetes Screeners
**Young adults with diabetes do not discuss DES/diabetes complications (B)** **Do (not) know other people their age with diabetes (M)** **Diabetes distress/burnout (B),** for example, a feeling of being overwhelmed by diabetes management	Social Role and Identity Social influences Emotion	Modelling Enablement Environmental restructuring	Social support (practical)^a^ Social support (emotional) Social comparison Credible source^a^ Information about others approval Problem solving^a^ Demonstration of the behaviour Framing/reframing Goal setting^a^ Self‐monitoring Action planning	Social media campaign including blogs and videos of YAs discussing their experience of attending DES. This could include ‘diabetes influencers’ or celebrities Peer support groups for YAs with diabetes organised by age. Groups could be facilitated by older YAs with diabetes who have experienced DES themselves. Could include facilitated discussion about DR/DES. Having YAs talk to each other about the issue, for example, reasons why do/do not attend, group problem solving and sharing of advice and tips, positive experiences. Offer YAs psychological support (e.g. counselling) Offer YAs psychological support (e.g. counselling) For diabetes distress/burnout, focus on emotional support, for example, focusing on one issue at a time by setting incremental goals. Provide tools to help with self‐monitoring, problem solving, action planning.	Communication /marketing targeted at YAs with diabetes YAs with diabetes Service level YAs with diabetes
**Preference to receive appointment information by text/e‐mail/phone call, instead of by letter (E)** **Forgetting to attend at least one DES appointment (B)** **Forgetting appointments because they are booked too far in advance (B)** **Putting the DES appointment in their electronic calendar once they receive the appointment letter (E)** **Impact of university and transitioning from paediatric to adult care (B)**	Memory, attention, decision‐making Behavioural Regulation	Enablement Training	Prompts/cues^a^ Instruction on how to perform behaviour^a^ Problem solving^a^ Social support (practical)^a^	Send appointment information using a range of modalities in addition to the appointment letter—that is, text message, phone (as letters not always received) Send additional reminders (i.e. prompts) for attendance closer to the date of the appointment (e.g. 1 week before), using a range of modalities—that is, text message, phone, letters Opportunity to set the date of next appointment at end of current appointment Deliver training which supports YA in developing strategies to remember appointments, for example, putting the appointment in their diary straight away, visible reminders (appointment letter on fridge, highlighted), asking a friend/family member to help remind you to attend etc. Encourage sharing of tips and strategies amongst YAs Make sure YA knows to inform the screening service of any change of address or change in registered GP practice	Service level YAs with diabetes

Note

Abbreviations: B, Barrier; DES, diabetic eye screening; E, Enabler; M, Mixed; TDF, Theoretical Domains Framework; YA, Young adult.

^a^ BCTs shown to be effective in Cochrane review of Interventions to improve DES.^19^

## 4 DISCUSSION

This study aimed to identify the barriers and enablers to DES attendance using the TDF to code the interviews, with an emphasis on modifiable behaviours. The key TDF domains in terms of frequency, elaboration and importance were *Knowledge*; *Social influences*; *Social role*/*identity*; *Environmental context*/*resources*; *Goals* and *Intention*. Many of the same theoretical domains were identified as barriers/enablers to DES in two previous studies using the TDF, including a study of young adults with type 2 diabetes in Australia^7^ and linguistic and cultural minority groups in Canada.^15^

Study participants included regular DES attenders, unintentional non‐attenders and those who had intentionally missed one or more screening appointments in the past. Many factors influencing behaviour were consistent across groups, for example, knowledge gaps regarding DES and its treatment (*Knowledge*), strong intentions to attend future DES appointments (*Intention*). Barriers more specific to non‐attenders included participants not knowing other people their age with diabetes, feeling ‘isolated’ and the ‘odd one out’ during school and teenage years and reluctance to disclose their diabetes (*Social role and identity*), diabetes distress/burnout and feeling nervous and anxious about receiving DES results (*Emotion*). Barriers more specific to unintentional non‐attenders included the lack of co‐ordinated diabetes care *(Environmental context and resources*) and lack of coordination between DES and other aspects of diabetes care (*Environmental context*/*resources*).

Young adults experience a range of contextual, practical and social challenges. First, they leave school, and often the parental home, may take a year out, for example, to travel, before entering higher education or the workplace. Young adults with diabetes must navigate these difficult transitions, whilst at the same time taking on increasing responsibility for their diabetes care.^20^ They may no longer have the necessary practical support and reminders from family members, which have been previously shown to be important enablers to attending DES.^6^, ^7^, ^15^ Study participants highlighted that the process of transitioning from paediatric to adult services was often associated with a failure to attend DES, with less frequent clinical appointments and being seen by an unfamiliar team. There were particular difficulties during the period of leaving home for university/college study and either having to change DES provider or being limited by the restriction to appointments outside term time. General difficulties with scheduling appointments, time demands associated with attending multiple clinical appointments, which are not coordinated and the negative effects of the eye drops were also seen as barriers for many study participants. The lack of appointment flexibility and the need to take time away from study or work were also reported as barriers amongst adolescents and young adults with type 1 diabetes in two recently published studies.^9^, ^10^

A particular issue in the United Kingdom is the separation of DES (which is managed as one of the five National Adult Population Screening Programmes) from other aspects of diabetes care. This lack of integration was reflected in the perceived communication difficulties between DES and other members of the diabetes care team. Furthermore, the physical separation between sites providing DES and other processes of care makes it difficult to integrate DES and screening tests for other diabetes complications. Improved communication and collaboration between the screening programme and GP practices^21^ associated with recommendations and reinforcement from HCPs to attend for DES^6^, ^7^, ^15^ have been identified as enablers for DES in previous studies. Another important potential role for GPs and other HCPs is to provide support and information after receiving DES results.

### 4.1 Implications for policy and practice

We have identified a range of potential strategies to increase DES attendance. Some interventions targeting the person with diabetes are relatively simple, for example, providing age‐appropriate information on the risk of developing retinopathy and its treatment and restructuring the content of results letters. A previous study investigating the effectiveness of a tailored evidence‐based information leaflet to promote uptake of DES in young adults with type 2 diabetes, found that this simple intervention significantly increased knowledge of diabetic retinopathy, an important predictor of DES uptake.^22^

Interventions directed at HCPs (e.g. GPs, diabetes team) could include the development of a nationally approved training programme that includes specific recommendations for actions HCPs could take to support, encourage and enable young adults to attend DES (e.g. how to raise the issue of DES and check screening attendance in a non‐judgemental way, how to facilitate referrals to DES services and how to provide reassurance and address concerns around diabetic retinopathy).

At a policy level, we have made recommendations to better integrate eye screening with other diabetes services. Although there is no currently high quality evidence from the United Kingdom that integrated ‘one‐stop clinics’ improve DRS uptake specifically in young adults, ‘collaborative case management’, which coordinates the processes of diabetes care, has been shown to improve diabetic retinopathy outcomes in trials of a general population of adults with diabetes.^23^

Another policy recommendation is to review the selective use of mydriatic drops in young adults. The National Screening Committee (NSC) currently recommends dilating all people attending for screening on the basis of the ease of organisation and improved image quality; however, there is evidence that targeted mydriasis strategies can be effective for DES.^24^

### 4.2 Strengths and limitations

One of the main strengths of the current study is that it addresses an important evidence gap and incorporated the views and experiences of young adults with diabetes in planning and conducting the research. Although there are many studies that have previously reported modifiable barrier/enablers to DES,^6^ these studies tended to treat people with diabetes as a homogeneous group, and therefore, it is not possible to identify barriers specific to particular population subgroups. Relatively few studies^7^, ^8^, ^9^, ^10^ have reported barriers from the perspective of young adults with diabetes and only two of these were based in United Kingdom.^6^, ^9^ Another strength of our approach is the use of a theory‐informed and replicable methodology to identify barriers and enablers.^11^ This provides a basis for generating evidence‐based change strategies (BCTs or programme changes) that are tailored to young adults to address barriers or enhance facilitators. A similar approach has been successfully adopted to increase DES uptake in a general population of people with diabetes in Ireland.^25^

Our inclusion criteria included young adults with type 1 and type 2 diabetes. Despite recruiting a diverse sample of people with type 1 diabetes in terms of demographic factors and screening behaviour, we were unable to recruit young adults with type 2 diabetes. Recruitment challenges in this population have been previously identified.^7^ The results of the current study therefore cannot be generalised to young adults with type 2 diabetes due to recognised clinical and psychosocial differences between type 1 and type 2 diabetes in this demographic group,^26^, ^27^ relative susceptibility to diabetic retinopathy^28^ and barriers to screening.^7^

Whilst the TDF provides a useful and comprehensive theoretical approach to identifying influences on behaviour, if applied too rigidly there is a risk that non TDF‐related factors could be missed.^29^ We attempted to mitigate this risk by using an inductive approach in the analysis to ensure that potential themes that could not be coded to the TDF were not lost.

### 4.3 Directions for future research

Type 2 diabetes is becoming increasingly prevalent in adolescents and young adults and further research is needed to evaluate strategies to increase their representation in health and medical research.

The results from the mapping of TDF domains to BCTs have identified a number of potentially effective target behaviours at multiple levels, many of which have been shown to be effective in a general population of people with diabetes^19^ Based on salient TDF domains and linked BCTs, we have proposed a number of potential intervention components that could be operationalised as part of a multicomponent strategy to increase young adult's DES attendance. Using a similar co‐design process to that described by Riordan et al.,^25^ the next step will be to discuss the acceptability and feasibility of the suggested intervention components. Once acceptability and feasibility have been considered they could be piloted and, if they meet a priori progression criteria, their effectiveness could be evaluated in a trial of these interventions to assess the impact on uptake in the target population.

## 5 CONCLUSIONS

The current study is the first in‐depth exploration of the factors influencing DES attendance from the perspective of UK young adults with type 1 diabetes. A behavioural approach was used, informed by the TDF, which allowed us to identify a number of barriers to and enablers of DES attendance. Although there were high levels of awareness of the importance of DES, there was a lack of knowledge of treatments available should diabetic retinopathy be detected. Many of the barriers related to the competing time demands and practical issues with scheduling DES appointments, including the lack of coordination with other aspects of diabetes care.

Identifying the theory‐informed determinants of DES attendance behaviour provides an opportunity to design interventions that specifically target these behaviours. It is likely that tailored approaches will be needed to facilitate implementation and uptake of DES in young adults. This study has identified a number of potential behavioural targets and programme changes that could be used to inform intervention components to address modifiable barriers and enhance enablers to attendance.

## CONFLICT OF INTEREST

None.

## DISCLAIMER

This report is independent research funded by the National Institute for Health Research (Policy Research Programme, Enabling diabetic RetinOpathy Screening: Mixed methods study of barriers and enablers to attendance (EROS study), PR‐R20‐0318–22001). The views expressed in this publication are those of the author(s) and not necessarily those of the NHS, the National Institute for Health Research or the Department of Health and Social Care.

## Supporting information

Supplementary Material
