# Supplementary material for: Barriers and enablers to diabetic eye screening attendance: An interview study with young adults with type 1 diabetes
Source: Diabet Med. 2021 Dec 29;39(3):e14751. doi: 10.1111/dme.14751 (PMC9304253; doi:10.1111/dme.14751)
Supplement: Supplementary file 1 — Supplementary Material [file DME-39-0-s001.docx]

**Supplementary Appendices**

**S1: Interview topic guide**

**TOPIC GUIDE – INTERVIEWS WITH YOUNG ADULTS WITH DIABETES**

**Introduction**

Thank you for speaking with me today. My name is Louise and I’m part of a research team at City, University of London. Can I please check that you still happy to take part in the interview?

We are interested in the views of young people with diabetes about going to diabetic eye screening. The interview will last approximately 40 minutes depending on how much you have to say.

I will audio record our discussion so that I don’t have to take as many notes and can listen to what you are saying. Do let me know if that is something you are not comfortable with. Following the interview, all identifying information mentioned will be removed from the transcript.

I am going to ask you a series of questions related to your thoughts about your diabetes and attending diabetes eye screening, and what stops or helps you to attend. I’m interested in your thoughts and experiences. You may find it uncomfortable to answer some of the questions. Please say if at any point you feel upset or distressed. The interview will be stopped and you will be given the option of speaking to a member of the research team with professional training in diabetic retinopathy, if you would like to. Remember that you are free to withdraw from the study at any time. Do you have any questions?

- ***[If written consent and demographic information have been obtained]***

Thank you for providing your written consent to take part in the study. Before we continue with the interview can I please check that you are still happy to proceed? Are you happy for me to start recording?

***[Start recording]***

**OR**

- ***[If taking verbal consent]***

Before we begin the interview, I need to record that you give your verbal consent to take part in the study. I will read out the contents of the consent form, which you also have a copy of. If you agree with the statements, please respond ‘I agree’. Please let me know if you have any questions as go through the form. Are you happy for me to start recording?

***[Start recording]***

***[Record verbal consent]***

I also need to ask you to provide some information about yourself. This will be used to describe who took part in our study as a whole. Everyone’s responses will be combined so you will not be identifiable.

***[Record demographic information]***

Are you happy to start the interview?

**Interview questions**

**Introductory questions**

1. **Can you tell me a bit about your diabetes?**

- Where are you seen?
- Which healthcare professionals do you see most regularly about your diabetes? (e.g. GP/diabetes nurse)

1. **Have you ever received any education or training about how to help manage your diabetes? [Skills]**

- If yes, did it cover diabetic eye screening?
- Is there anything else you would like to know?

1. **On a scale of 1-10 how engaged are you with managing your diabetes?**

**[Behavioural regulation]**

**Move onto diabetes and the eyes**

1. **Have you heard that diabetes can affect your eyes? [Knowledge]**

**If yes:**

- Can you talk me through what have you heard?
- Have you heard of diabetic retinopathy?
  - If yes, where did you learn about it? From whom?
  - Do you know the symptoms of diabetic retinopathy?

*[If no, ‘Diabetic retinopathy occurs when diabetes affects small blood vessels in the area at the back of the eye, called the retina. It can cause the blood vessels in the retina to leak or become blocked. This can affect your sight’.]*

1. **Is diabetic retinopathy a concern for you? [Emotions]**

- Previously (if affected)
- At present?
- In the future? **[Optimism]**
- How often do you think about diabetic retinopathy?
  - Are there times you think more/less about it?

1. **What is the purpose of attending diabetic eye screening? [Knowledge]**

- Can you talk me though the aims/purpose?
- Do you know what diabetic eye screening involves?
- How often should you attend?

*[If no, ‘In the UK, everyone with diabetes aged 12 years and over is offered annual diabetic eye screening. Screening involves having eye drops put into your eyes, to make the pupils temporarily larger. Then photographs of the back of the eye (retina) will be taken to look for early signs of diabetic retinopathy that might need treatment. Diabetic eye screening can detect diabetic retinopathy before you notice any changes to your vision.]*

1. **Do you know what treatments are available if diabetic retinopathy is detected? [Knowledge/Beliefs about consequences]**

- What do you think about these?

1. **Do you know other people your age with diabetes? [Social influences]**

- If yes,
  - How many people?
  - Do you spend time with them often?
  - Do you know if they typically attend diabetic eye screening?
- Have you seen anything related to diabetic eye screening on social media?
- Has this/have these influenced your decision to attend diabetic eye screening appointments?

1. **How do you feel about telling people you have diabetes? [Social professional role/ identity]**

- Why would you not tell someone about it?
- Has this influenced your decision to attend diabetic eye screening appointments?

1. **Are you in the habit of attending your diabetic eye screening appointments? [Behavioural regulation]**

- How often?
- Tended to go consistently?
- Any periods of not attending?
- If not, what would help to make it more routine?

1. **How are you made aware of your diabetic eye screening appointments? [Memory attention decision making]**

- What do you think of this/these methods?
- How much notice do you receive?
- Has there ever been an occasion where you weren’t notified about an appointment? (Moving from home/transition paediatric to adult services)

1. **Have you ever decided not to attend an eye screening appointment?**

- **If yes, why?**

1. **Have you ever forgotten to attend a diabetic eye screening appointment?**

**[Memory attention decision making]**

- What helps/might help you to remind you to attend a diabetic eye screening appointment?

1. **Where do your diabetic eye screening appointments take place?**

**[Environmental context and resources]**

- How easy/difficult are the appointments for you to get to and from?

(probe rural/city location) **[Beliefs about capabilities]**

- How do these fit in with other diabetes appointments you might need to attend?

1. **Have you ever encountered any problems getting to and from the screening appointments in the past? [Behavioural regulation]**

- Do you require any assistance to attend the appointments?

1. **Typically, how much time do the diabetic eye screening appointments take out of your day? (including after effects of eye drops/getting to the appointment)**

**[Environmental context and resources]**

- How does this fit in with your other commitments?
- How convenient are the time and dates of the appointments?
  - Are the appointment date/times flexible?
- Has this influenced your decision to attend diabetic eye screening appointments?

1. **Is attending diabetic eye screening a high or low priority in terms of your diabetes management? [Goals]**

- Why?
- What else is high/low priority?
- How does it fit into your life more generally?

1. **What are your reasons for attending? [Beliefs about consequences]**

- Are there any drawbacks of attending?
- To what extent do the reasons outweigh the drawbacks?

1. **[If attend/sometimes attend] How does attending the diabetic eye screening appointments make you feel? [Emotion]**

- Do you have any concerns?
- How do you feel when you get the results?

1. **[Non-attenders] How does not attending the diabetic eye screening appointments make you feel?**

- How do you think attending the diabetic eye screening appointments would make you feel?

1. **How long do you have to wait for your test results?**

- How are the test results communicated to you?
- Do you receive any support or advice following the test results?

1. **Have you received any support or encouragement to attend your diabetic eye screening appointments? [Social influences]**

- If yes, who from? (Friends, family, healthcare professionals, social media)
  - How did the encouragement support you attending?
- If no, what kind of encouragement might support you attending?

1. **Do you ever feel any pressure to attend eye screening appointments?**

**[Social influences]**

- **Where from?** (Friends, family, healthcare professionals, social media)

1. **How likely do you think it is that you will attend your eye screening appointments in the future? [Intentions]**

- Why?
- Why not?

1. **Is there anything that would make you more likely to start/continue attending the eye screening appointments? [Reinforcement]**
2. **How have you found taking part in this interview [signpost to Diabetes UK, JDRF if necessary].**

Thank you for your time, your responses are very helpful. Is there anything else that I haven’t covered or you would like to add? Do you have any questions?

**S2. Coding framework**

**Supplementary Table 1.EROS Data Analysis Codebook – Young Adults**

| **Domain** | **Construct** | **Decision rule** | **Example** |
| --- | --- | --- | --- |
| 1. **Knowledge**   *An awareness of the existence of something* | **Knowledge (including knowledge of condition/scientific rationale):** An awareness of the existence of something  **Procedural knowledge:** Knowing how to do something  **Knowledge of task environment:** Knowledge of the social and material context in which a task is undertaken. | Consider coding to this domain:  Discussion about broader diabetes management  Discussion about how diabetes affects the eyes (link between the two)  Discussion about the purpose of attending DES  Awareness of the symptoms of diabetic retinopathy  Awareness of treatment for diabetic retinopathy  Procedural knowledge: Awareness of what DES involves e.g. taking a photo of the back of the eye  Awareness of screening guidelines e.g. attending once a year from the age of 12 years  ***Could show high/low levels of knowledge in answers***  ***Include other peoples’ awareness of DES e.g. HCPs***  Inappropriate coding to this domain:  Reasons for attending/not attending – Consider coding to Beliefs about Consequences  Description of symptoms experienced – Consider coding to MADM  Discussion of from whom they learnt about diabetic retinopathy – Consider coding to Social Influences | *So high blood sugars or large variations can cause problems eventually in your sight with your blood vessels and stuff? P5F1*  *[…] you can get damage in the back of your eye related to having high blood glucose levels which can cause, it can cause eye damage or blindness. P26F1*  *Yeah, because I think a lot of people just don’t really understand it and they don’t really know the purpose of it [DES]. P15F1*  *So they check the back of your eyes. They look at the back of your eyes to see if they can see any kind of damage there. P26F1* |
| 1. **Skills**   *An ability or proficiency acquired through practice* | **Skills:** An ability or proficiency acquired through training and/or practice  **Skills development:** The gradual acquisition or advancement through progressive stages of an ability or proficiency acquired through training and practice  **Competence:** One’s repertoire of skills, and ability especially as it is applied to a task or set of tasks  **Ability:** Competence or capacity to perform a physical or mental act. Ability may be either unlearned or acquired by education and practice  **Interpersonal skills:** An aptitude enabling a person to carry on effective relationships with others, such as an ability to cooperate, to assume appropriate social responsibilities or to exhibit adequate flexibility  **Practice:** Repetition of an act, behaviour, or series of activities, often to improve performance or acquire a skill  **Skills assessment:** A judgement of the quality, worth, importance. Level or value of an ability or proficiency acquired through training and practice | Consider coding to this domain:  Courses/training which cover diabetes management  Courses/training which cover DES  Any further information/training the participant would like to know about diabetes management/DES  Inappropriate coding to this domain:  Descriptions of how easy/difficult it is for the participant to manage their diabetes/attend DES – Consider coding to Beliefs about Capabilities  Descriptions of how confident the participant is they can manage their diabetes/attend DES – Consider coding to Beliefs about Capabilities | *It probably did. It would’ve been brief though. In, yeah, a couple of things that I’ve been to talk about it maybe for ten minutes. Yeah. [Do you remember if that covered diabetic eye screening at all?] P5F1* |
| 1. **Social/professional role and identity**   *A coherent set of behaviours and displayed personal qualities of an individual in a social or work setting* | **Professional identity:** The characteristics by which an individual is recognised relating to, connected with or befitting a particular profession  **Professional role:** The behaviour considered appropriate for a particular kind of work or social position  **Social identity:** The set of behavioural or personal characteristics by which an individual is recognizable [and portrays] as a member of a social group  **Identity:** An individual’s sense of self defined by a) a set of physical and psychological characteristics that is not wholly shared with any other person and b) a range of social and interpersonal affiliations (e.g., ethnicity) and social roles.  **Professional boundaries:** The bounds or limits relating to, or connected with a particular profession or calling  **Professional confidence:** an individual’s belief in his or her repertoire of skills and ability especially as it is applied to a task or set of tasks. Group identity: the set of behavioural or personal characteristics by which an individual is recognizable [and portrays] as a member of a group  **Leadership:** The processes involved in leading others, including organising, directing, coordinating and motivating their efforts toward achievement of certain group or organization goals  **Organizational commitment:** An employee’s dedication to an organisation and wish to remain part of it. Organisational commitment is often described as having both an emotional or moral element and a more prudent element | Consider coding to this domain:  Discussion about which HCP (role) they see most often about their diabetes/DES  Discussion about how the participant feels disclosing their diabetes to other people  Discussion about how many other young adults they know with diabetes  Discussion about membership with diabetes groups e.g. charity youth groups  Inappropriate coding to this domain:  Discussion of support provided by HCP – Consider coding to Social Influences | *Yeah during school, [Less comfortable telling people about diabetes] because where I had it from a young age, you don’t want to be the odd one out. P3M1* |
| 1. **Beliefs about capabilities**   *Acceptance of the truth, reality, or validity about an ability, talent or facility that a person can put to constructive use* | **Self-confidence:** Self-assurance or trust in one’s own abilities, capabilities and judgement  **Perceived competence:** An individual’s belief in her or her ability to learn and execute skills  **Self-efficacy:** An individual’s capacity to act effectively to bring about desired results, as perceived by the individual  **Perceived behavioural control:** an individual’s perception of the ease or difficulty of performing the behaviour of interest  **Beliefs:** The thing believed; the proposition or set of propositions held true  **Self-esteem:** The degree to which the qualities and characteristics contained in one’s self concept are perceived to be positive  **Empowerment:** The promotion of the skills, knowledge and confidence necessary to take great control of one’s life as in certain educational or social schemes; the delegation of increase decision-making powers to individuals or groups in a society or organization  **Professional confidence:** An individual’s beliefs in his or her repertoire of skills, and ability, especially as it is applied to a task or set of tasks | Consider coding to this domain:  Descriptions of how easy or difficult it is for the participant to manage their diabetes  Descriptions of how confident a participant feels that they can manage their diabetes  Descriptions of how easy or difficult it is for the participant to attend DES  Descriptions of how confident a participant feels that they can attend DES  Inappropriate coding to this domain:  Descriptions of how confident a participant feels that they can tell other people about their diabetes – Consider coding to Social/professional role and identity | *It was more difficult, [To attend appointments when caring for parents] but I managed. P3M1* |
| 1. **Optimism**   *The confidence that things will happen for the best or that desired goals will be attained* | **Optimism:** The attitude that outcomes will be positive and that people’s wishes or aims will be ultimately fulfilled  **Pessimism:** The attitude that things will go wrong and that people’s wishes or aims are unlikely to be fulfilled  **Unrealistic optimism:** the inert tendency for humans to over-rate their own abilities and chances of positive outcomes compared to those of other people | Consider coding to this domain:  Participants’ descriptions of their levels of optimism regarding management of their diabetes  Participants’ descriptions of their levels of optimism regarding developing diabetic retinopathy  ***Code both positive and negative answers*** | *Not in that I think that I’m going to get it (inaudible). [Is diabetic retinopathy a concern for you?] P4F1* |
| 1. **Beliefs about consequences**   *Acceptance of the truth, reality or validity about outcomes of a behaviour in a given situation* | **Beliefs:** The thing believed; the proposition or set of propositions held true  **Outcome expectancies:** Cognitive, emotional, behavioural, and affective outcomes that are assumed to be associated with future or intended behaviour. These assumed outcomes can either promote or inhibit future behaviours.  **Characteristics of outcome expectancies:** Characteristics of the cognitive, emotional and behavioural outcomes that individuals believe are associated with future or intended behaviours and that are believed to either promote or inhibit these behaviours. These include whether they are sanctions/rewards, proximal/distal, valued/not valued, probable/improbable. Salient/not salient, perceived risks or threats.  **Anticipated regret:** A sense of the potential negative consequences of a decision that influences the choice made: for example an individual may decide not to make an investment because of the feelings associated with an imagined loss    **Consequents:** An outcome behaviour in a given situation | Consider coding to this domain:  Consequences of diabetes self-management behaviours and development of DR  Consequences of attending DES appointments e.g. early detection of symptoms  Consequences of not attending DES appointments e.g. miss changes in the eye  Potential long-term consequences of attending DES appointments e.g. maintain eye health and vision  Potential long-term consequences outcomes of not attending DES appointments e.g. sight loss  Outcomes of the screening procedure e.g. Mydriatic drops = uncomfortable, not being able to drive  ***Can include advice given to participants from HCP***  ***Code both positive and negative answers***  Inappropriate coding to this domain: Concerns about DR as a condition. Consider coding to Emotion  Discussion about the cause of DR as a condition in general – Consider coding to Knowledge | *Just to look after my eyes. To watch out for anything going wrong. [Reason for attending appointments] P5F1*  *So my last test came back as having background retinopathy, because, and I did ask my doctor about it, and she said that if you have a HbA1c of 7 or above for about ten years then it's natural to get some background, but it shouldn't affect my eyesight, I think. P8F1* |
| 1. **Reinforcement**   *Increasing the probability of a response by arranging a dependent relationship, or contingency, between the response and a given stimulus* | **Rewards (proximal/distal, valued/ not valued, probable/improbable):** Return or recompense made to, or received by a person contingent on some performance  **Incentives:** An external stimulus, such as condition or object, that enhances or serves as a motive for behaviour  **Punishment**: The process in which the relationship between as response and some stimulus or circumstance results in the response becoming less probable; a painful, unwanted or undesired event or circumstance imposed as a penalty on a wrongdoer  **Consequents:** An outcome of behaviour in a given situation  **Reinforcement:** A process in which the frequency of a response is increased by a dependent relationship or contingency with a stimulus  **Contingencies:** A conditional probabilistic relation between two events. Contingencies may be arranged via dependencies or they may emerge by accident  **Sanctions:** A punishment or other coercive measure, usually administered by a recognized authority that is used to penalise and deter inappropriate or unauthorized actions. | Consider coding to this domain:  Reinforcement/reward of attending DES  Consider:   - Financial incentives - Praise - Past experiences - Intrinsic reward e.g. reassurance (also Emotion) - Punishment   *It is likely that this domain will come up less often than the others  Inappropriate coding to this domain:  Discussion of social reinforcement  (e.g. support from family/HCP) -Consider coding to Social Influences | *So, it does reassure me that whatever I’m doing is, is working. P2F1* |
| 1. **Intention**   *A conscious decision to perform a behaviour or a resolve to act in a certain way* | **Stability of intentions:** ability of one’s resolve to remain in spite of disturbing influences  **Stages of Change model:** A model that proposes that behaviour change is accomplished through five specific stages  **Transtheoretical model and stages of change:** a five-stage theory to explain changes in people’s health behaviour. It suggests that change takes time, that different interventions are effective at different stages, and that there are multiple outcomes occurring across the stages | Consider coding to this domain:  Participant’s descriptions of how motivated they are to attend DES  Participant’s descriptions of when they are more or less inclined to attend DES  Participant’s descriptions of what might make others more or less inclined to attend DES  Participant’s inclinations to attend all of their DES appointments  Inappropriate coding to this domain:  This is different from the benefits of attending DES (‘Beliefs About Consequences’) and different from how much of a priority attending DES is for them (‘Goals’).  Be careful not to code the reasons for the intention (focus on statements that directly reflect their intention and motivation) | *I will just go. [Is there anything that would make you more likely to continue going? Or do you think you would just go already?] P3M1*  *I know for other people it might be helpful to have some more encouragement. You know like, even once they send the letter if that came with a leaflet about the importance of the screening or something or a bit more information about it. Because then it makes it feel a bit more real or something, because I know some people will just brush it aside. P15F1* |
| 1. **Goals**   *Mental representations of outcomes or end states that an individual wants to achieve* | **Goals (distal/proximal):** Desired state of affairs of a person or system, these may be closer (proximal) or further away (distal)  **Goal priority:** Order of importance or urgency of end state toward which one is striving  **Goal/target setting:** A process that establishes specific time-based behavioural targets that are measureable, achievable and realistic  **Goals (autonomous/controlled):** The end state toward which one is striving: the purpose of an activity or endeavour. It can be identified by observing that a person ceases or changes their behaviour upon attaining this state; proficiency in a task to be achieved within a set period of time.  **Implementation intention:** The plan that one creates in advance of when, where and how one will enact a behaviour | Consider coding to this domain:  Attending DES as a high/low priority in terms of participant’s diabetes management  Other aspects of participant’s diabetes management which are a high/low priority  Inappropriate coding to this domain:  This is different from the benefits of diabetes management/attending DES (‘Beliefs About Consequences’)  Clear examples of action planning – Consider coding to Behavioural Regulation | *Yeah, so high, just keeping, probably keeping my blood sugar stable. Keeping active and stuff like that. [What other parts of the diabetes management would you say are a high priority?] P5F1* |
| 1. **Memory, attention and decision processes**   *The ability to retain information, focus selectively on aspects of the environment and choose between two or more alternatives* | **Memory:** The ability to retain information or a representation of a past experience, based on the mental processes of learning or encoding retention across some interval of time, and retrieval or reactivation of the memory; specific information of a specific task  **Attention:** A state of awareness in which the senses are focussed selectively on aspects of the environment and the central nervous system is in a state of readiness to respond to stimuli  **Attention control:** The extent to which a person can concentrate on relevant cues and ignore all irrelevant cues in a given situation  **Decision making:** The cognitive process of choosing between two or more alternatives, ranging from the relatively clear-cut to the complex  **Cognitive overload/tiredness:** The situation in which the demands placed on a person by mental work are greater than a person’s mental abilities | Consider coding to this domain:  Discussion about DR symptoms participant has experienced    Discussion about how frequently participant thinks about DR  Discussion about how the participant is made aware of their DES appointments  Discussion about the participant forgetting to make/attend their DES appointments  Discussion about the participant weighing up the pros and cons of attending DES appointments  Discussion about appointment reminders e.g. text/phone call  Inappropriate coding to this domain:  Awareness of DR symptoms – Consider coding to Knowledge  Discussion around how attending the appointment makes the participant feel – Consider coding to Emotion | *I just get them [notification about appointment] through the post actually. P26F1*  *Yeah, [Nurse/Consultant asking about appointment is helpful] because it could possibly slip my mind. I mean it probably wouldn’t because I’m quite on top of going to them. So, but I guess for people that weren’t, so it might be helpful. P4F1* |
| 1. **Environmental context and resources**   *Any circumstance of a person’s situation or environment that discourages or encourages the development of skills and abilities, independence, social competence, and adaptive behaviour* | **Environmental stressors:** External factors in the environment that cause stress  **Resources/material resources:** Commodities and human resources used in enacting a behaviour    **Organizational culture/climate:** A distinctive pattern of thought and behaviour shared by members of the same organization and reflected in their language, values, attitudes, beliefs and customs  **Salient events/critical incidents:** Occurrences that one judges to be distinctive, prominent or otherwise significant  **Person x environment interaction:** Interplay between the individual and their surroundings  **Barriers and facilitators:** In psychological contexts, barriers/facilitators are mental, emotional or behavioural limitations/strengths in individuals or groups | Consider coding to this domain:  Discussion around the type of clinic they attend e.g. young adult clinic  Discussion about how DES appointments fit in with participant’s other routine diabetes appointments  DES service configuration e.g. do they have their DES appointments in the same hospital every time  Discussion around the ease/difficulty of getting to and from the DES appointments  -Whether the DES appointments are in a rural or urban location and how this impacts attendance/travel  Discussion about how DES appointments fit in with participant’s other commitments e.g. employment, childcare, other immoveable life events  Scheduling appointment issues e.g. long wait/not receiving an appointment/insufficient notice  Discussion about waiting times and the length of the DES appointments  Discussion about how the participant is made aware of the results  Discussion around support available following receipt of results e.g. phone number to call  Financial concerns e.g. costs owing to lost income  Discussion around availability of resources related to diabetes management/DES e.g. leaflets, online resources  Inappropriate coding to this domain:  Discussion around how much of a priority attending DES is within their overall diabetes management/life – consider coding to Goals  Discussion around social support e.g. family member driving participant to appointment – Consider coding to Social Influences | *I go to the clinic maybe two times a year to see the consultants, and it’s like a young adult’s clinic I attend. P15F1*  *Yeah, very convenient because it’s usually in the morning, about 8, 9 o’clock. P3M1*  *I don’t see the same person, but I go to the same place. P2F1*  *I think there’s a phone number you can call if you want to ask any more questions or anything. P15F1*  *I think it’s [information about DES] available if you look for it but it’s not really something that is really publicised. P15F1* |
| 1. **Social influences**   *Those interpersonal processes that can cause individuals to change their thoughts, feelings, or behaviours* | **Social pressure:** the exertion of influence on a person or group by another person or group  **Social norms:** Socially determined consensual standards that indicate a) what behaviours are considered typical in a given context and b) what behaviours are considered proper in the context  **Group conformity:** The act of consciously maintaining a certain degree of similarity to those in your general social circles  **Social comparisons:** The process by which people evaluate their attitudes, abilities or performance relative to others  **Group norms:** Any behaviour, belief, attitude or emotional reaction held to be correct or acceptable by a given group in society  **Social support:** The apperception or provision of assistance or comfort to others, typically in order to help them cope with a variety of biological, psychological and social stressors. Support may arise from any interpersonal relationship in an individual’s social network, involving friends, neighbours, religious institutions, colleagues, caregivers of support groups  **Power:** The capacity to influence others, even when they try to resist this influence Intergroup conflict: Disagreement or confrontation between two or more groups and their members. This may involve physical violence, interpersonal discord, or psychological tension.  **Alienation:** Estrangement from one's social group; a deep-seated sense of dissatisfaction with one's personal experiences that can be a source of lack of trust in one's social or physical environment or in oneself; the experience of separation between thoughts and feelings    **Group identity:** the set of behavioural or personal characteristics by which an individual is recognizable [and portrays] as a member of a group  **Modelling:** In developmental psychology the process in which one or more individuals or other entities serve as examples (models) that a child will copy | Consider coding to this domain:  How other people react to them having diabetes e.g. social stigma  Other people’s lack of awareness/understanding of diabetes e.g. difference between T1/T2  Influence of social media e.g. do they see much related to DES?  Recommendation from HCP to attend DES e.g. do diabetes team check if they have attended?  Encouragement/support participant receives from family to attend DES  Encouragement/support participant would like to receive from HCP/family to attend DES  Inappropriate coding to this domain:  Discussion about how the participant feels disclosing their diabetes to other people – Consider coding to Social/professional role and identity  Discussion about which HCP (role) they see most often about their diabetes/DES – Consider coding to Social/professional role and identity  Discussion about awareness of the purpose off attending DES – Consider coding to Knowledge | *It’s obviously sometimes difficult because people don’t really understand the difference between Type 1 and Type 2 diabetes and people just don’t really understand it in general […] P15F1*  *They would just check in and ask really, just like ask you if you’ve been attending them [DES appointments]. P15F1*  *And maybe it would be nice to speak to a consultant because I remember when I first started having some problems at the start of the year it was a meltdown. ‘Oh my goodness, this is the worst thing in the whole world’, but you’d no consultant to speak to about it. So yeah, that wasn’t so good. P5F1* |
| 1. **Emotion**   *A complex reaction pattern, involving experiential, behavioural and physiological elements, by which the individual attempts to deal with a personally significant matter or event* | **Fear:** An intense emotion aroused by the detection of imminent threat, involving an immediate alarm reaction that mobilizes the organism by triggering a set of physiological changes  **Anxiety:** A mood state characterized by apprehension and somatic symptoms of tension in which an individual anticipates impending danger, catastrophe or misfortune.    **Affect:** An experience or feeling of emotion, ranging from suffering to elation, from the simplest to the most complex sensations of feelings, and from the most normal to the most pathological emotional reactions.    **Stress:** A state of physiological or psychological response to internal or external stressors  **Depression:** A mental state that presents with depressed mood, loss of interest or pleasure, feelings of guilt or low self-worth, disturbed sleep or appetite, low energy, and poor concentration  **Positive/negative affect:** the internal feeling/state that occurs when a goal has/has not been attained. A source of threat has/has not been avoided, or the individual is/is not satisfied with the present state of affairs Burn-out: Physical, emotional or mental exhaustion, especially in one’s job or career, accompanied by decreased motivation, lowered performance and negative attitudes towards oneself and others | Consider coding to this domain:  Distress/burnout from managing diabetes (general emotional state)  Fear/anxiety about diabetic retinopathy diagnosis  Fear/anxiety about DES procedure  Fear/anxiety receiving DES results  Reassurance when receive DES results (also reinforcement)  Discussion about times when participant worries more/less about DR e.g. when blood sugars are high  ***Code both positive and negative answers***  Inappropriate coding to this domain:  Concerns about how capable participant is at attending DES appointments – Code to Beliefs about Capabilities  (Long-term) outcomes of attending/not attending DES – Code to Beliefs about Consequences | *So I’ve literally been probably two years in the past just really burnt out with and overwhelmed by diabetes care. P2F1*  *Fine. I’m not really nervous or anything like that about them [Attending the appointments]. I just get on with it. P4F1*  *It’s normally a relief if they’re OK, but it’s nice getting the results as well just to know that there’s nothing going wrong. P15F1*  *I think if you ever have a period of when your blood sugars are really high or their just not working the way you want them to and then you just get really frustrated with yourself and you start worrying about all the things that might happen if they keep going that way. I think that’s the times that I freak out more about the eyes and feet and everything to do with that. P15F1* |
| 1. **Behavioural regulation**   *Anything aimed at managing or changing objectively measured actions* | **Self-monitoring:** A method used in behavioural management in which individuals keep a record of their behaviour, especially in connection with efforts to changes or regulate the self; a personality trait reflecting an ability to modify one’s behaviour in response to a situation  **Breaking habit:** to discontinue a behaviour or sequence of behaviours that is automatically activated by relevant situational cues  **Action planning:** The action or process of forming a plan regarding a thing to be done or a deed. | Consider coding to this domain:  Engagement with diabetes management  Engagement with DES appointment attendance  Clear examples of action planning  Things participant does to make attending the appointments easier e.g. making it part of a habitual routine | *You’d have to, [Get people to drive her to appointments in remote location] my mum would take me. P5F1* |

**S3. Full list of themes (i.e. barriers/enablers) identified within each domain**

**Supplementary Table 2.Themes identified within each TDF domain**

| **TDF domain** | **Theme** | **Representative quotation** | **Barrier/ enabler/mixed** | **Frequency** | **RA**  **INA**  **UNA** | **Spontaneous (Y/N)** | **Potentially modifiable (Y/N)** |
| --- | --- | --- | --- | --- | --- | --- | --- |
| **Knowledge** | 1. Understanding how diabetes affects the eyes | “So, from what I know, it's that if you've got a regular high sugar levels, the vessels at the back of your eye burst and sort of leak, and that affects your vision and you get patches.” (PF81) | **E** | **23:** All except 3 4 12 13 14 17 | 11 RA  6 INA  6 UNA | N | Y |
|  | 1. (Not) understanding the reasons for attending DES | “I think it’s just they can just check to see if there’s any changes in your eyes so from the pictures and if they notice anything different then they can follow it up as soon as it happens instead of waiting until it gets worse.” (P15F1)  “I do now, but I never used to […] I didn’t feel that I was given any education on why my screenings were important in the first year or two. So I went to my first one, didn’t go to my second.” (P7M1) | **M** | **26:**  All except 10 11 16  **3:**  1 7 18 | 13 RA  8 INA  5 UNA  1 RA  2 INA  0 UNA | N | Y |
|  | 1. Not knowing a lot about the treatments available if DR is detected | “I don’t really know much, I just know that either they keep an eye on it for a few years, or you keep going in to see a specialist instead of the regular screenings and then if it gets worse I think it starts with the laser and then a couple of operations can be done as well I think, but I don’t really know that much about what’s done.” (P10F1) | **B** | **22**: All except 7 12 13 14 18 19 23 | 12 RA  5 INA  5 UNA | N | Y |
|  | 1. Not understanding how to prevent the development of DR | “What can I do now to make sure that I don’t get retinopathy in the future, rather than just trying to spot it? I know it [DES] is prevention […] but they could probably go even further than that and say, rather than actually screening, make sure that your blood sugar levels don’t go to X, Y or Z.” (P16M1) | **B** | **4:**  2 5 9 16 | 2 RA  0 INA  2 UNA | Y | Y |
|  | 1. Awareness of diabetes and DES | “I think retinopathy is more of a sensitive topic or it’s probably the most sensitive topic when it comes to diabetic complications, because it can just go completely untreated, and then all of a sudden the person has real issues. So, I think the people doing the screening should have a little bit more understanding and empathy that, of what diabetes is and the nuances.” (P1M1)  “I think maybe just a bit more awareness of it, you know if a leaflet came out or something with the letter or even if there was more on social media about it. It might just make me more aware of the effects of if I don’t go to these screenings.” (P15F1) | **M** | **6:**  1 7 10 11 12 18 25  **3:** 1 7 15 | 5 RA  2 INA  0 UNA  2 RA  1 INA  0 UNA | Y | Y |
|  | 1. Understand that managing blood sugars lessens chance of developing DR | “[…] hopefully if I keep them that way [blood sugars good] when I’m older then I shouldn’t get any problems.” (P6F1) | **E** | **5:**  6 8 11 25 28 | 4 RA  0 INA  1 UNA | Y | Y |
|  | 1. Understanding that people with diabetes experience more complications as they get older | “The older you get, the more problems you’re going to get with it I suppose.” (P14M1) | **E** | **3:**  10 14 16 | 1 RA  1 INA  1 UNA | Y | Y |
| **Skills** | 1. Receipt of education/training about how to manage diabetes | “I’ve done the DAFNE course which is obviously around carb counting.” (P7M1) | **E** | **24:** All except 8 9 12 26 29 | 13 RA  6 INA  5 UNA | N | Y |
|  | 1. Education/training didn’t cover DES in detail | “It probably did. It would’ve been brief though. In, yeah, a couple of things that I’ve been to talks about it maybe for ten minutes.” (P5F1) | **B** | **20:** All except 6 8 9 12 16 20 22 26 29 | 11 RA  7 INA  2 UNA | N | Y |
| **Social/professional role and identity** | 1. Do (not) know other people their age with diabetes | “So, in my area when I was younger, they used to send us off on diabetic camp.” (P10F1)  “No. I don’t know anyone.” (P26F1) | **M** | **22**: 1 2 4 6 7 10 11 12 13 14 15 16 17 18 19 20 21 22 23 24 25 29  **7:** 3 5 8 9 26 27 28 | 13 RA  5 INA  4 UNA  2 RA  3 INA  2 UNA | N | Y |
|  | 1. Feeling ‘isolated’ and ‘the odd one out’ during school/teenage years | “[…] just going through the teenage years was really difficult, just trying to fit in at school and manage Type 1 […] I would say it’s pretty isolating because you feel like you’re going through everything on your own.” (P18M1) | **B** | **7:** 3 12 13 16 18 25 29 | 3 RA  3 INA  1 UNA | Y | N |
|  | 1. Confidence in disclosing diabetes | “I don’t mind at all because I like to educate them a bit if they don’t know anything.” (P2F1)  “Very reluctant, I’m renowned for being somebody who keeps it quite secret, until I know somebody well.” (P23F1)  “At first, when I was six years old, I felt a bit weird talking about it but, as I’ve got older, I’ve just accepted it.” (P16M1) | **M** | **13:** 1 2 4 6 9 11 12 15 19 20 21 22 28  **6:** 5 8 10 23 24 26  **9:** 3 7 13 14 16 17 18 27 29 | 10 RA  1 INA  2 UNA  2 RA  1 INA  3 UNA  2 RA  6 INA  1 UNA | N | N |
|  | 1. Knowing others with diabetes/being part of the online community means you are more engaged with diabetes management | “Yeah as I've got older, as I've got more in touch with it, as, I think one of the key things was again back to this point about seeing other peoples’ experiences and realising that the often portrayed solution to diabetes being carbs plus insulin equals blood sugar is incredibly misleading, in terms of its complexity and once I understood that and really came to peace with that and it wasn’t just I was crap at counting or it wasn’t somehow my fault that this formula didn’t work, that I think is really what then empowered me to feel like I could take control of everything else.” (P27M1) | **E** | **5:** 10 18 25 27 29 | 2 RA  3 INA  0 UNA | Y | Y |
|  | 1. Positive impact of technology on diabetes self-management | “So, I recently just got a FreeStyle Libre, what four, five days ago, and my blood sugar management within the four, five days has just dramatically improved.” (P12M1) | **E** | 7: 7 12 16 23 24 25 29 | 3 RA  2 INA  2 UN | Y | Y |
| **Beliefs about capabilities** | 1. Having well controlled diabetes/blood sugars | “I am really well controlled, so I don’t really need to see them [DSN, diabetes specialist] or speak to them that much.” (P10F1) | **E** | **13:** 1 5 6 7 9 10 11 12 16 17 19 25 28 | 9 RA  1 INA  3 UNA | Y | N |
| **Optimism** | N/A – No themes |  |  |  |  |  |  |
| **Beliefs about consequences** | 1. Negative impact of eye drops | “I just remember obviously coming out afterwards and I was walking down the corridor and I just couldn’t see a thing and I couldn’t see my phone to ring my grandma or anything. So I was, I know so I was walking down I must have looked like an absolute crazy person.” (P24F1) | **B** | **9:** 2 3 7 8 11 13 24 25 29 | 4 RA  3 INA  2 UNA | Y | N |
|  | 1. Attend DES to avoid DR, to monitor eyes and for the early detection of complications | “So, I am trying to obviously go to my [DES] appointments and stuff like that, so that I can avoid getting it [DR] […] Because I don’t want to be end up not being able to see.” (P4F1) | **E** | **17:** 4 5 8 10 11 13 14 15 16 18 19 21 22 23 27 28 29 | 8 RA  4 INA  5 UNA | N | Y |
|  | 1. Long-term consequences of not engaging with diabetes care as an enabler to DES attendance | “I know that I’ve got the issues with my eye, so I need to go there to make sure that everything’s OK and I understand the reasons why.” (P18M1) | **E** | **2:** 3 18 | 0 RA  2 INA  0 UNA | Y | N |
|  | 1. Emphasising importance of attending DES suggested as way of encouraging YA to attend | “I know for other people it might be helpful to have some more encouragement. You know like, even once they send the letter if that came with a leaflet about the importance of the screening or something or a bit more information about it. Because then it makes it feel a bit more real or something, because I know some people will just brush it aside.” (P15F1) | **E** | **3**: 10 12 15 | 3 RA  0 INA  0 UNA | Y | Y |
|  | 1. Importance of eyesight | “[DR a concern] Because I do believe it’s, my eyes are really, really valuable to me. I read a lot.” (28M1) | **E** | **5:** 8 11 20 24 28 | 2 RA  1 INA  2 UNA | Y | N |
| **Reinforcement** | 1. Mixed feelings of pressure to attend DES | “No, no pressure, no.” (P20F1)  “Only pressure that I put on myself.” (P7M1) | **M** | **17:** 1 2 3 4 5 6 9 15 17 20 21 22 24 25 26 28 29  **3:** 7 18 19 | 10 RA  4 INA  3 UNA  0 RA  2 INA  1 UNA | N | Y |
| **Intention** | 1. Strong intention to attend future DES appointments | “Very likely. I don't intend to miss any of them.” (P13F1) | **E** | **27:** All except 19 20 | 15 RA  7 INA  5 UNA | N | Y |
|  | 1. Pregnancy as a life event increases intention to attend DES appointments | “When I was pregnant, I had them every other month because they keep a really close eye on you when you’re pregnant. I knew that there was more chance of something happening when I was pregnant and, so, I would, yeah, definitely go to them.” (P2F1) | **E** | **1:** 2 | 1 RA  0 INA  0 UNA | Y | N |
| **Goals** | 1. Priorities in diabetes management | “I think for my diabetes management, I think the main thing for me is literally just managing my glucose levels and, day to day […] it’s my diet and stuff like that. Staying fit and making sure that I don’t get any complications further down the line with retinopathy or heart disease or anything associated with that.” (16M1) | **E** | **25:** All except 1 2 11 18 | 12 RA  7 INA  6 UNA | N | Y |
|  | 1. Attending DES becomes more of a priority when experienced complications | “If I hadn’t had that past experience, if someone said to me, [participant name], it’s going to take five hours out of your day or, I’d probably go, (sighs) well I’ll go next week and then I’d probably forget about it.” (P1M1) | **E** | **6:** 1 3 8 14 22 24 | 2 RA  2 INA  2 UNA | Y | N |
|  | 1. ‘All diabetes appointments’ are seen as high priority | “Well I try to keep my sugars under good control as much as possible, so it’s quite high priority for it all really, to be honest.” (P14M1) | **E** | **6:** 3 4 13 14 25 27 | 3 RA  3 INA  0 UNA | Y | N |
| **Memory, attention and decision processes** | 1. Forgetting to attend at least one DES appointment | “I’ve never actively chosen not to. I think I just might have forgotten to go to it but I’ve never […] it’s not a case of, I’m not getting any value from this.” (P16M1) | **B** | **9:** 3 5 7 8 16 18 19 21 26 | 0 RA  4 INA  5 UNA | N | Y |
|  | 1. Thinking about DR when screening appointments are due or when blood sugars are high | “I would say I probably think about it more when my blood sugars are running higher. So that’s usually when I’m quite stressed or anxious generally and if my blood sugars are running higher and I’ve noticed that they’re running higher I do worry about it and think about it more than I would normally.” (P21F1) | E | **10:** 1 3 4 5 6 8 16 17 21 28 | 5 RA  1 INA  4 UNA | N | Y |
|  | 1. Prompts and reminders to attend | “Probably a text [would have helped remind her to attend], because people do check their phones more often now I think. So I think a text reminder would have helped.” (P8F1) | **E** | **10:** 1 2 3 5 8 11 12 16 18 23 | 5 RA  2 INA  3 UNA | N | Y |
|  | 1. Preference to receive appointment information by text/e-mail/phone call, instead of by letter | “I travel quite a lot with work so there might be times where I miss the letter, or my parents, I live with my parents and they’re terrible for letting letters pile up and it will get put to the bottom of that pile [….] so if they were to send a letter, an email or a text message, or even just give me call that would be really useful.” (P7M1) | **E** | **8:** 1 7 11 16 24 26 28 29 | 3 RA  3 INA  2 UNA | N | Y |
|  | 1. Forgetting appointments because they are booked too far in advance | “Probably (laughs) yeah probably [has forgotten to attend DES appointments in the past]. They be sending them quite far in advance, the letters. So if you don’t keep on top of it, it’s quite easy, you’d forget.” (P5F1) | **B** | **2:**5 8 | 0 RA  0 INA  2 UNA | Y | Y |
|  | 1. Receive enough notice for DES appointments | “Yeah, usually. The last one I went to we had to rearrange it but there was enough time in advance to rearrange the date and everything and I still got to go to the appointment.” (P15F1) | **E** | **10:** 6 10 11 12 13 15 18 20 21 25 | 7 RA  2 INA  1 UNA | N | Y |
|  | 1. Delayed diagnosis as a barrier to screening attendance | “I actually had, when I, by the time I was diagnosed I actually had deterioration of my eyes already because it took me so long to get diagnosed.” (P20F1) | **B** | **5:** 12 17 20 21 28 | 3 RA  1 INA  1 UNA | Y | N |
| **Environmental Context & Resources** | 1. DES appointments are (not) easy to get to | “Very easy. It’s just one bus from outside my house, all the way there.” (P3M1)  “I used to live in the very north of [country name] and you’d have to travel 120 miles to get to any appointment. So that wasn’t ideal […] There’s a lack of availability up there […] It probably did impact it [DES attendance] really.” (P5F1) | **M** | **19:** 1 3 4 5 6 7 8 13 15 17 18 19 20 21 22 23 25 27 29  **8:** 5 9 10 14 16 24 25 26 | 9 RA  7 INA  3 UNA  3 RA  3 INA  2 UNA | N | Y |
|  | 1. DES and diabetes care are (not) co-ordinated | “They’re just, all my appointments are just random. Nothing lines up it’s all just, they just come at random.” (P20F1)  “Usually pretty well actually. They dovetail quite nicely in between my consultant appointments.” (P27M1) | **M** | **12:** 5 6 8 9 10 14 16 19 20 21 22 26  **5:** 3 13 23 27 28 | 4 RA  3 INA  5 UNA  3 RA  2 INA  0 UNA | N | Y |
|  | 1. DES fits in with studying as it is flexible | “They fit in fine, I think. Obviously for the past 8 years I've been a student most of the time so I haven't really had a day where I've thought I can't attend this appointment, usually I just go to them, so they fit in quite well I think.” (P8F1) | **E** | **7**: 5 8 15 17 16 21 28 | 3 RA  0 INA  4 UNA | N | N |
|  | 1. Need for more flexibility and options for (re-)scheduling DES appointments | “Just another option of day, just a, yeah, just a second option just to say, look this is the other day that we can do. Just so that I can make the best decision for me around my schedule.” (P7M1) | **B** | **14:** 1 6 7 9 10 11 16 18 21 22 24 25 26 29 | 7 RA  4 INA  3 UNA | Y | Y |
|  | 1. Advantages of not having mydriatic drops | “If you could find a way to see the back of your eye without having to have the drops so you could drive. No, that’s everything. I could go first thing in the morning before work then.” (P2F1) | **E** | **8:** 2 5 6 7 15 17 21 28 | 4 RA  1 INA  3 UNA | Y | Y |
|  | 1. DES appointments take up half a day or more | “[…] if you include the drops, probably three to four hours until your eyesight’s fully back to normal.” (P29M1) | **B** | **14:** 1 2 3 9 10 14 16 19 20 23 24 25 27 29 | 6 RA  5 INA  3 UNA | N | Y |
|  | 1. Employers (not) accommodating to DES appointments | “No, my work’s very good, they’re very easy. I can just go out and get it done and come back.” (P6F1)  “Because I work on an interim bases so if I don’t work I am not paid. I am paid for the hours I do.” (P9F1) | **M** | **9:** 4 6 7 10 13 14 18 22 27  **5:** 9 20 24 26 29 | 5 RA  4 INA  0 UNA  1 RA  1 INA  3 UNA | Y | Y |
|  | 1. Having to take leave for DES appointment or make time back at work | “I used to work quite far away from there. So, it would mean that I had to take maybe even a day off […] I’d rather just do it in my spare time when I wouldn’t have to take leave.” (P16M1) | **B** | **5:** 2 14 16 20 28 | 2 RA  2 INA  1 UNA | Y | Y |
|  | 1. Impact of Covid-19 on DES attendance | “I was due to have one there in April and I still haven't had it yet because of the coronavirus and again they're I think six months behind anyway. So I haven’t had it this year at all.” (P13F1) | **B** | **4:** 9 10 13 20 | 3 RA  1 INA  0 UNA | Y | N |
|  | 1. Impact of university and transitioning from paediatric to adult care | “Yes, there was a period of time somewhere around the transition, maybe even at university actually and the transition out of university where I didn’t go because I'd just fallen off the list a bit and at that point wasn’t really engaged with it.” (P27M1) | **B** | **6:** 10 13 16 18 22 27 | 3 RA  2 INA  1 UNA | Y | Y |
| **Social influences** | 1. Mixed views about level of DR related content on social media | “Only the stuff I’ve seen from you with your research, I have to say [DR related posts on social media].” (P23F1)  “So, yeah, just seeing people going through various treatments on social media.” (P26F1) | B | **17:** 1 6 8 9 10 11 13 14 15 16 17 19 21 23 24 25 29  **7:** 2 4 7 12 18 26 28 | 10 RA  2 INA  5 UNA  4 RA  3 INA  0 UNA | N | Y |
|  | 1. Need for more support and information following DES results | “[…] you don’t need someone saying like you’re doing a shit job. What you need is […] you need someone to explain to you, OK, this is what we found, or this is what potentially could happen. These are the options, don’t worry about it, do you know what I mean?” (P1M1) | **B** | **12:** 1 5 9 11 12 13 16 20 22 26 27 29 | 6 RA  4 INA  2 UNA | Y | Y |
|  | 1. Diabetes team check DES appointment attendance | “Well, every time I go for my meeting, I go every six months, and every time I go, they ask when I last had my eyes screened. And then, if I haven’t had it done, they’ll send off, they’ll ask for an appointment then.” (P2F1) | **E** | **13**: 2 4 6 10 11 15 16 18 19 21 27 28 29 | 7 RA  3 INA  3 UNA | N | Y |
|  | 1. Partners/family members assist in getting to and from their DES appointments | “So, I would have my mum or my girlfriend give me a lift and it’s just a bit impractical sometimes.” (P16M1) | **E** | **14:** 1 2 4 5 7 8 13 14 16 20 22 23 24 25 | 7 RA  3 INA  4 UNA | N | Y |
|  | 1. Young adults with diabetes don’t discuss DES/diabetes complications | “I do think as well just that, when it comes to the diabetes community that you don’t always talk about complications, so that’s not the nice side of living with Type 1. So, you can get peer support, but you don’t always find people who are openly talking about their complications.” (P18M1) | **B** | **6**: 1 13 16 18 21 29 | 2 RA  1 INA  3 UNA | Y | Y |
|  | 1. Impact of healthcare professional communication | “When I got told I had the macular oedema thing my GP was like, you’re a bit young to have any diabetic complications, and it’s like, well I have been diabetic 20 years, that’s the same as someone getting it when they’re 40 and seeing complications when they’re 60, it’s just, yeah. So, I think there’s a bit of a gap where even medical professionals don’t really think about what they’re saying sometimes.” (P10F1)  “I think for me, there’s something about knowing that I’ve got a team behind me, that’s there to help me that makes me think, I want to attend that, I want to be there, I want to be part of it. I think that’s a huge focus for me, so just that communication between a diabetic and whoever’s the healthcare professionals, I think it’s just really important.” (P12M1) | **M** | **7:** 1 6 9 10 13 18 23  **7:** 1 9 12 17 18 24 25 | 6 RA  1 INA  0 UNA  5 RA  1 INA  1 UNA | Y | Y |
|  | 1. Seeing older people with worse complications in the DES waiting room | “I think awareness in the sort of environment you going to […] I find that every time I go it’s just full of really old people that have not necessarily taken the best care of their diabetes. So a lot of the time I’ll go and sit there and there’ll be people without their legs and stuff and I just find it not very pleasant to sit in the waiting room and look around.” (P10M1) | **B** | **3:** 10 16 22 | 2 RA  0 INA  1 UNA | Y | Y |
|  | 1. Learning about diabetes management/DES from family | “And also my dad has Type 2 Diabetes, and he has screening as well, so I think I probably was aware of it, because he went to a screening appointment prior to me being diagnosed, I think, so it was known.” (P23F1) | **E** | **3:** 5 9 23 | 2 RA  0 INA  1 UNA | Y | N |
| **Emotion** | 1. DR is a concern | “Yeah [DR a concern] I think everyone worries that they’re going to lose, well, not everyone but if there’s a possibility you’re going to lose your sight, it’s going to be a bit of a worry.” (P2F1) | **E** | **19:** 1 2 3 5 7 8 9 10 11 13 14 15 16 20 21 25 27 28 29 | 9 RA  6 INA  4 UNA | N | Y |
|  | 1. Worry about future complications as a result of diabetes | “I want to live as good a life as anyone else. And I think the potential consequences, the potential threats of what diabetes can cause, not looking after your sugar levels with potential problems in your feet and your extremities and in your eyesight as well, that scares me and I just want to do everything I can really to be normal.” (P25M1) | **E** | **10:** 1 7 10 12 13 15 16 20 21 25 | 6 RA  2 INA  2 UNA | Y | Y |
|  | 1. Diabetes distress/burnout | “So I’ve literally been probably two years in the past just really burnt out with and overwhelmed by diabetes care and slipped into a pattern of just not taking my medication, not checking my blood glucose levels and not going to eye screening so, yeah.” (P26F1) | **B** | **4:** 18 20 25 26 | 1 RA  3 INA  0 UNA | Y | Y |
|  | 1. Diabetes scare stories | “So, yes, so I was diagnosed when I was ten, so it was when I’m 12/13 I was hearing, well if you don’t look after yourself, this is going to happen, if you don’t look after yourself you know you can get heart disease, you can get kidney failure, and you’re telling that to, in effect, a child.” (P18M1) | **B** | **6:** 8 10 13 18 22 25 | 4 RA  1 INA  1 UNA | Y | Y |
|  | 1. Mixed feelings about receiving DES results | “Yeah, I feel fine. It’s good to have peace of mind, I guess.” (P17F1)  “It depends on if they’re good or bad I’d say. Yeah, usually not great about it to be honest.” (P26F1) | **M** | **5:** 2 15 17 20 21  **13:** 1 3 7 8 9 10 11 18 19 24 25 26 28 | 3 RA  1 INA  1 UNA  6 RA  4 INA  3 UNA | N | N |
| **Behavioural Regulation** | 1. Putting DES appointments in electronic calendar once they receive the appointment letter | “I just put it in my calendar now […] a little thing in my Google calendar, yeah.” (P16M1) | **E** | **3:** 1 3 16 | 1 RA  1 INA  1 UNA | Y | Y |

**S4. Full list of suggested intervention strategies**

**Supplementary Table 3.Candidate intervention strategies to improve DES**

|  | **Identified barrier (B)/enabler (E)/mixed theme (M)** | **Corresponding TDF domain** | **Intervention function (Behaviour Change Wheel)** | **Behaviour Change Technique** | **Proposed operationalisation of selected intervention components** |
| --- | --- | --- | --- | --- | --- |
| **1** | **(Not) understanding the reason for attending DES (M) –** some participants previously did not understand the reasons for attending DES  **Not knowing a lot about the treatments available if DR is detected (B)**  **DR is a concern (E)** – main reason cited - fear of sight loss in the future | Knowledge  Emotion  Beliefs About Consequences | Education  Persuasion | *Information about health consequences  Salience of consequences  BCTs not wanting to deliver: anticipated regret. Need to put emphasis on the positives to minimise negative emotions rather than prompting feelings of anxiety and regret  Framing/re-framing  *Credible source | Providing information on: 1) risks of developing DR and risks of progression using contemporary data, 2) potential complications if DR goes undetected. Emphasis placed on positive rather than negative information to minimise defensive or avoidant responses - e.g. emphasise the benefits of early detection (pick things up early + can be treated).  Providing information on available treatments for DR - emphasise again the positives - e.g. effectiveness of treatments in helping to stop DR from progressing (particularly if caught early)  Leaflet, a social media campaign etc. Case studies/testimonials (e.g. video, digital resources) by other YAs with diabetes that demonstrate positive emotions and outcomes as a result of screening (i.e. I attended my screening regularly, and this meant as soon as any small changes were picked up, they could be treated straight away to stop them progressing into something more serious or sight threatening) - instead of testimonials that focus on the extremes- i.e. I lost my vision/went blind because I left it too late  Reinforcement by screeners that attending DES reduces the risk of vision loss and YAs don’t need to live in fear of future blindness  Communication on reasons for attending and available treatments with HCPs (e.g. GP, Optometrist, Diabetologist) |
| **2** | **Awareness of diabetes and DES (B)** - a lack of understanding amongst the general public and HCPs  **Impact of healthcare professional communication (M)**  **Diabetes scare stories (B) -** participants told by HCPs about the potential negative impact of diabetes  **Diabetes team check DES appointment attendance (E)** | Knowledge  Social Influences | Education  Training  Persuasion  Enablement | *Information about health consequences  *Instruction on how to perform the behaviour  Demonstration of the behaviour  Behavioural practice/rehearsal  *Credible source  Information about emotional consequences  Anticipated regret  Salience of consequences  Feedback on behaviour | Health information campaigns to promote awareness about both diabetes and DES, including the importance of attending DES, younger people having diabetes, and the difference between type 1 and type 2 diabetes. This could include social media campaigns, leaflets, TV adverts, talks in schools and colleges etc.  For HCPs (e.g. GPs, diabetes team) – develop nationally approved training delivered by accredited HCPs using educational material adjusted for different age-groups. This should include specific recommendations for actions HCPs can take to support, encourage and enable YAs to attend DES (e.g. how to raise the issue of DES and check screening attendance in a non-judgmental way, how to facilitate referrals and access to convenient DES services, how to provide reassurance and address concerns around DR, complications/sight loss, and DES, how to reinforce the benefits of screening). Training could include videos demonstrating a HCP speaking to a YA with diabetes, showing how to raise the issue sensitively. Plus, opportunities for HCPs completing the training to practice/role playing different communications. Training could include a testimonial from a YA with diabetes describing how negative communication impacted them detrimentally  Provide HCPs feedback/ data on DES attendance in local area. Highlighting the low attendance rates in YAs (to persuade GPs that this is an issue/ draw their attention to it). |
| **3** | **Education/training didn’t cover DES in detail (B)** | Skills  Knowledge | Training  Education  Persuasion  Enablement | *Instruction on how to perform the behaviour  *Information about health consequences  *Credible source  *Problem solving | Include a session specifically on DES in diabetes self-management programmes - this should ideally cover the link between diabetes and vision, the purpose and importance of screening (again with an emphasis on positives), with patient testimonials, and any advice or tips on how to enable DES attendance (e.g. strategies for helping to remember/arrange DES, how to discuss with HCPs etc.). This could also include opportunities for people attending the training to think about potential barriers to attending DES and problem solve around potential solutions (as often group-format courses)  Role for paediatric/transition clinics in education and training |
| **4** | **Confidence in disclosing diabetes (M)** -YAs reluctant to disclose/discuss their diabetes with other people | Social/Professional Role and Identity | Modelling  Education | Identity associated with changed behaviour  Framing/reframing  Demonstration of the behaviour  *Instruction on how to perform the behaviour  Information about social and environmental consequences  *Social support (practical)  Social support (emotional)  *Credible source  Information about others approval | Encourage YAs create a positive identity associated with diabetes e.g. something they shouldn’t feel ashamed of, but should feel empowered to talk about on their own terms. This could be done by providing examples from others/persuasive strategies/messages  Increase/provide observable examples of YAs disclosing/discussing their diabetes with a mix of others (e.g. friends, family, colleagues/ employers, healthcare providers) e.g. through storylines on TV, social media peer support groups  Suggested scripts/wording/advice tips/ examples of how YAs with diabetes can disclose their diabetes to others  Education in schools on difference between type 1 and type 2 diabetes  Educate YAs significant others (e.g. family, close friends, partners) about diabetes management. This could include in-person education sessions held at the hospital and/or information leaflets/booklets e.g. ‘how to support your family member with diabetes’. Could also include advice on things they can do to support people with diabetes following disclosure. |
| **5** | **Young adults with diabetes don’t discuss DES/diabetes complications (B)**  **Do (not) know other people their age with diabetes (M)**  **Diabetes distress/burnout (B)** e.g. a feeling of being overwhelmed by diabetes management | Social/Professional Role and Identity  Social influences  Emotion | Modelling  Enablement  Environmental restructuring | *Social support (practical)  Social support (emotional)  Social comparison  *Credible source  Information about others approval  *Problem solving  Demonstration of the behaviour  Framing/reframing  *Goal setting  Self-monitoring  Action planning | Social media campaign including blogs and videos of YAs discussing their experience of attending DES. This could include ‘diabetes influencers’ or celebrities  Offer YAs psychological support (e.g. counselling)  Peer support groups for YAs with diabetes organised by age. Groups could be facilitated by older YAs with diabetes who have experienced DES themselves. Could include facilitated discussion about DR/DES. Having YAs talk to each other about the issue e.g. reasons why do/do not attend, group problem solving and sharing of advice and tips, positive experiences.  For diabetes distress/burnout, focus on emotional support e.g. focusing on one issue at a time by setting incremental goals. Provide tools to help with self-monitoring, problem solving, action planning. |
| **6** | **Preference to receive appointment information by text/e-mail/phone call, instead of by letter (E)**  **Forgetting to attend at least one DES appointment (B)**  **Forgetting appointments because they are booked too far in advance (B)**  **Putting the DES appointment in their electronic calendar once they receive the appointment letter (E)**  **Impact of university and transitioning from paediatric to adult care (B)** | Memory, Attention, Decision Making  Behavioural Regulation | Enablement  Training | *Prompts/cues  *Instruction on how to perform behaviour  *Problem solving  *Social support (practical) | Send appointment information using a range of modalities in addition to the appointment letter – i.e. text message, phone (as letters not always received)  Send additional reminders (i.e. prompts) for attendance closer to the date of the appointment (e.g. 1 week before), using a range of modalities – i.e. text message, phone, letters  Opportunity to set the date of next appointment at end of current appointment  Deliver training which supports YA in developing strategies to remember appointments e.g. putting the appointment in their diary straight away, visible reminders (appointment letter on fridge, highlighted), asking a friend/family member to help remind you to attend etc.  Encourage sharing of tips and strategies amongst YAs  Make sure YA knows to inform the screening service of any change of address or change in registered GP practice |
| **7** | **DES and diabetes care are (not) co-ordinated (M)** | Environmental Context and Resources | Environmental Restructuring  Enablement | Restructuring the physical environment | Integrating eye screening with other diabetes services (e.g. ‘one-stop shop’ clinics). Less likely to forget and easier to arrange and monitor attendance and progress.  Improving integration by facilitating communication between provider teams (i.e. automatic transfer of results/screening attendance between primary care, secondary care, and DES service) |
| **8** | **Employers (not) accommodating to DES appointments (M)**  **Need for more flexibility and options for (re) scheduling DES appointments (B)**  **DES appointments take up half a day or more (B)**  **Seeing older people with worse complications in the DES waiting room (B)** | Environmental Context and Resources | Environmental restructuring  Enablement | *Problem solving  *Instruction on how to perform behaviour  Restructuring the physical environment | Encourage YAs to identify barriers to attending DES within the workplace, and identify strategies. E.g. - a barrier may be that they have not disclosed their diabetes to their manager, therefore, cannot request time off for diabetes related appointments. Offer them support/example solutions as to how to address barriers that might come up often e.g. how to raise the issue with employers and how to frame the request for time off. *(Relevant to employers (not) accommodating to DES appointments only)*  Increase availability of DES appointments to improve flexibility and choice in scheduling. In particular, include the provision of evening/weekend appointments  Allow self-booking of appointments  Dedicated DES clinics for YAs |
| **9** | **DES appointments are (not) easy to get to (M)** | Environmental Context and Resources | Environmental Restructuring  Enablement | Restructuring the physical environment  *Social support (practical) | Provide screening within the community, in more accessible locations e.g. mobile screening vans or in town centres/near shops,  Hand-held cameras in GP clinics  Having a family member or friend drive person with diabetes to/from appointments |
| **10** | **Need for more support and information following DES results (B)**  **Mixed feelings about receiving DES results (M)**  **Feeling nervous/anxious about receiving DES results** | Social Influences  Emotions | Environmental Restructuring  Enablement  Training | *Instruction on how to perform behaviour  Demonstration of the behaviour  Behavioural practice/rehearsal    Restructuring the physical environment  Social support (unspecified)  *Information about health consequences  Reduce negative emotions | Screeners who are suitably qualified providing initial indication of the likely result at DES appointment, so YAs don’t have to wait 2-3 weeks for results letter. This may involve providing further training for screeners, and sample scripts/videos of how to discuss the results in an appropriate way  Restructuring the content of the results letters so that the test result is accompanied by a clear explanation of the results (in lay language), reassurance about treatment options (phrased/framed positively), and contact details for people YAs can discuss the results with (e.g. consultant/GP/someone from the DESP)  Educational material to contain explanation about the findings and advice given.  Online support with FAQs*.* |
| **11** | **Attending DES becomes more of a priority when experienced complications (E)** | Goals | Persuasion  Education  Modelling | Information about health consequences  Credible source  Framing/reframing | Social media campaigns + leaflets with testimonials from people with diabetes who benefitted from attending DES. Emphasis on a positive frame – focused on the benefits of early detection rather than the ‘left it too late’ testimonials aiming to prompt regret. |
| **12** | **Negative impact of eye drops (B)** | Beliefs About Consequences  Environmental Context and Resources | Persuasion  Education  Environmental restructuring  Enablement | *Information about health consequences  *Credible source  Adding objects to the environment  Restructuring the physical environment | Provide information about possible after effects of the eye drops – so YAs are prepared. Emphasise the reasons for eye drops and their effects being short term vs the benefits of DES. This could include case studies/testimonials from other YAs with diabetes describing how the drops affected them  Improve signage in locations where DES is delivered (e.g. hospitals, health centres)  Limiting the use of the eye drops to only the individuals who really need them. This would reduce barriers to DES attendance including: time taken out of day, the unpleasant after effects of the drops, and travel to and from the appointment |

DR = diabetic retinopathy; DES = diabetic retinopathy screening; YAs = young adults; HCPs = healthcare professionals
